# Lerf–Klinowski-type models of graphene oxide and reduced graphene oxide are robust in analyzing non-covalent functionalization with porphyrins

**DOI:** 10.1038/s41598-021-86880-1

**Published:** 2021-04-12

**Authors:** Alexandra Siklitskaya, Ewelina Gacka, Daria Larowska, Marta Mazurkiewicz-Pawlicka, Artur Malolepszy, Leszek Stobiński, Bronisław Marciniak, Anna Lewandowska-Andrałojć, Adam Kubas

**Affiliations:** 1 Institute of Physical Chemistry, Polish Academy of Sciences, Kasprzaka 44/52, 01-224 Warsaw, Poland; 2 Faculty of Chemistry, Adam Mickiewicz University, Uniwersytetu Poznanskiego 8, 61-614 Poznan, Poland; 3 Center for Advanced Technology, Adam Mickiewicz University, Uniwersytetu Poznanskiego 10, 61-614 Poznan, Poland; 4 Faculty of Chemical and Process Engineering, Warsaw University of Technology, Warynskiego 1, 00-645 Warsaw, Poland; 5 NANOMATERIALS Leszek Stobinski (www.nanomaterials.pl), Warsaw, Poland

**Keywords:** Theoretical chemistry, Graphene, Electronic properties and devices, Physical chemistry, Optical spectroscopy

## Abstract

Graphene-based nanohybrids are good candidates for various applications. However, graphene exhibits some unwanted features such as low solubility in an aqueous solution or tendency to aggregate, limiting its potential applications. On the contrary, its derivatives, such as graphene oxide (GO) and reduced graphene oxide (RGO), have excellent properties and can be easily produced in large quantities. GO/RGO nanohybrids with porphyrins were shown to possess great potential in the field of photocatalytic hydrogen production, pollutant photodegradation, optical sensing, or drug delivery. Despite the rapid progress in experimental research on the porphyrin-graphene hybrids some fundamental questions about the structures and the interaction between components in these systems still remain open. In this work, we combine detailed experimental and theoretical studies to investigate the nature of the interaction between the GO/RGO and two metal-free porphyrins 5,10,15,20-tetrakis(4-aminophenyl) porphyrin (TAPP) and 5,10,15,20-tetrakis(4-hydroxyphenyl) porphyrin (TPPH)]. The two porphyrins form stable nanohybrids with GO/RGO support, although both porphyrins exhibited a slightly higher affinity to RGO. We validated finite, Lerf–Klinowski-type (Lerf et al. in J Phys Chem B 102:4477, 1998) structural models of GO ($\hbox {C}_{59}\hbox {O}_{26}\hbox {H}_{26}$) and RGO ($\hbox {C}_{59}\hbox {O}_{17}\hbox {H}_{26}$) and successfully used them in ab initio absorption spectra simulations to track back the origin of experimentally observed spectral features. We also investigated the nature of low-lying excited states with high-level wavefunction-based methods and shown that states’ density becomes denser upon nanohybrid formation. The studied nanohybrids are non-emissive, and our study suggests that this is due to excited states that gain significant charge-transfer character. The presented efficient simulation protocol may ease the properties screening of new GO/RGO-nanohybrids.

## Introduction

Ever since the explosion of interest to graphene following its successful fabrication in 2004^1–5^ the graphene functionalization remains a hot topic in the field. Graphene is a two dimensional (2D) single layer of graphite, that possesses unique mechanical, optical and electrical properties such as superconductivity and high Young’s modulus^1^. Functionalization of this novel material broadened the physicochemical properties ranges and the field of possible applications^6^. However, pure graphene exhibits very low solubility in water and tend to aggregate due to $\pi$–$\pi$ interactions. This limits some of its potential applications. For this reason, most known approaches to design composite materials rely on graphene derivatives such as graphene oxide (GO) and reduced graphene oxide (RGO)^7,8^. Both species have tunable properties and can form stable aqueous suspensions. GO is typically produced by chemical exfoliation of graphite through strong oxidization and is widely considered as an individual sheet of graphene decorated with various oxygen-containing functional groups (such as hydroxyl, epoxy, and carboxyl)^9,10^. The chemical reduction of GO leads to RGO with partly restored structure and properties of pure graphene, depending on the reduction process applied^11,12^. Among various graphene-based materials, the dye-functionalized graphenes are attractive candidates for nanohybrid platforms with peculiar photoactive properties.

The synthesis of nanohybrids involving non-covalent interactions between GO or RGO and dyes have important advantages, such as high yields, facile synthetic routes and maintenance of the main properties of each component^13–16^. Non-covalent chemical modification of GO or RGO with dyes is based on fundamental concept of molecular interactions, such as electrostatic attraction, $\pi$–$\pi$ stacking and hydrogen bonding. Porphyrins were chosen as a representable and extensively studied group of organic dyes with excellent spectroscopic and electrochemical properties. They are characterized by remarkably high extinction coefficients in the visible region. Thanks to their ability to transfer an electron as the result of photoexcitation, they may act as photosensitizers^17^.

Scientific community’s deep interest in porphyrin- and graphene-based hybrid materials is reflected in a number of reports^16,18–25^. Up to now, several groups have reported on improved photocatalytic activity of porphyrin/graphene nanohybrids toward pollutant photodegradation^22–25^. Yuan et al.^20^ presented enhanced photocatalytic activity toward hydrogen generation in a non-noble metal system for photocatalytic $\hbox {H}_2$ generation that combined Zn(II)-5,10,15,20-tetrakis(4-*N*-methylpyridyl) porphyrin and RGO decorated with $\hbox {MoS}_{2}$ as the catalyst. Porphyrin/graphene-based materials found application in optical^21^ and biological^26–28^ sensing, drug delivery^29^ or cancer therapy^16^.

In spite of the rapid progress in experimental research on the porphyrin-graphene hybrids some fundamental questions about the structures and the interaction between components in these systems still remain open. Elucidation of the mechanism of the interaction between the graphene materials and porphyrins including electronic properties of these materials is crucial for knowledge-driven design of nanomaterials and devices with desired properties^6^. Surprisingly, in spite of all these recent advances in the field of porphyrin/graphene nanoassemblies there are just few reports that combine experimental and theoretical investigations of such materials^30–35^.

GO and RGO are amorphous systems and as such constitute challenge to any computational protocol. Periodic density functional theory (DFT) calculations most often assume some degree of repeating order in oxygen atoms distribution on graphene sheets^36–39^. Other approach focus on local interactions of the adsorbate with graphene-derivative surface that is modeled as a graphene sheet decorated with very few oxygen-containing groups^40^. Out of various finite structural models proposed over the years^41–43^, the model of Lerf et al.^44^ (denoted as Lerf–Klinowski model, LK) was explicitly or implicitly assumed in many successful functionalization studies^43^. It contains $sp^{2}$ and $sp^{3}$ hybridized carbon atoms and various substituents such hydroxy, epoxy or carboxylic groups. Its size restricted many previous studies to force-field based methodologies. Despite such simplified treatment of atomic interactions, these were very successful in describing the dynamics of GO/water interface^7,45,46^. We have recently demonstrated the use of a finite, LK-based $\hbox {C}_{59}\hbox {O}_{26}\hbox {H}_{26}$ model to study graphene oxide interactions with selected porphyrins^30,31^. On one hand side, the model allows for detailed analysis of frontier molecular orbitals thus provides basis for spectra interpretation^30^. On the other hand, we noted that it may be used for efficient absorption spectra simulation of zinc-substituted porphyrins^31^.

**Figure 1:**
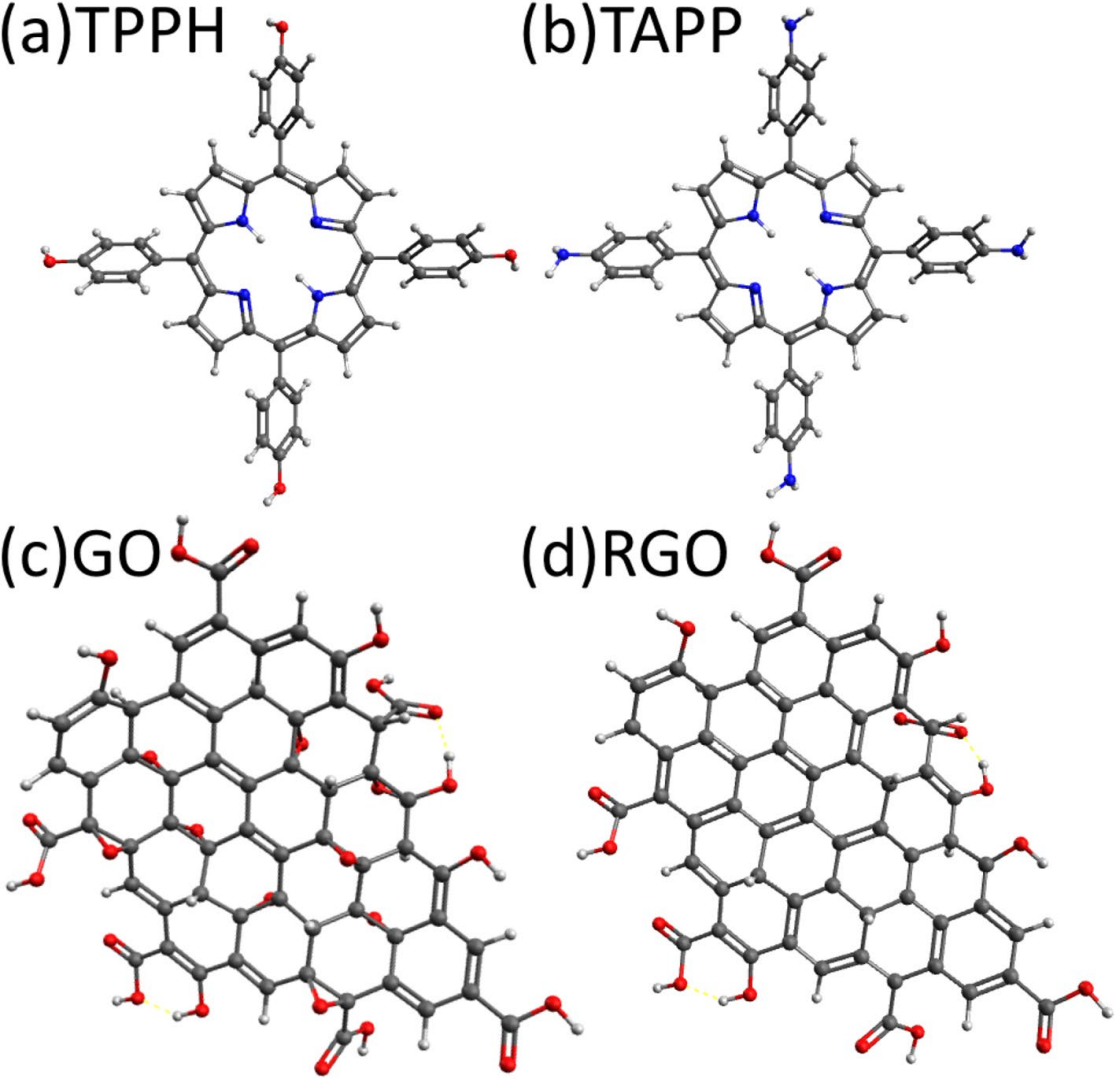
Optimized structures of (**a**) TPPH, and (**b**) TAPP molecules as well as the graphene derivatives used in this study: (**c**) graphene oxide (GO), and (**d**) reduced graphene (RGO).

The main aim of this study is to explore the robustness of the proposed LK-based models to describe nanohybrids’ formation processes. We focus on the nanoassemblies of two porphyrins, 5,10,15,20-tetrakis(4-aminophenyl) porphyrin (TAPP) and 5,10,15,20-tetrakis(4-hydroxyphenyl) porphyrin (TPPH) with GO and RGO (Fig. 1). GO is represented as $\hbox {C}_{59}\hbox {O}_{26}\hbox {H}_{26}$ structure^30,31^ while appropriate RGO model $\hbox {C}_{59}\hbox {O}_{17}\hbox {H}_{26}$ was generated from parent GO by gradual change of C/O ratio as described in the Computational details. The successful validation of the proposed models rely on the combination of experimental and theoretical approaches. We investigated the nature of the interaction between the components and described the influence of GO or RGO on the spectroscopic properties of porphyrin. Subsequently, we reconstructed the local GO and RGO structures as well as their complexes with TAPP and TPPH and modelled their experimental properties such as absorption spectra in the visible region and the density of the electronically excited states.

## Results and discussion

### Experimental studies

#### Absorption

**Figure 2:**
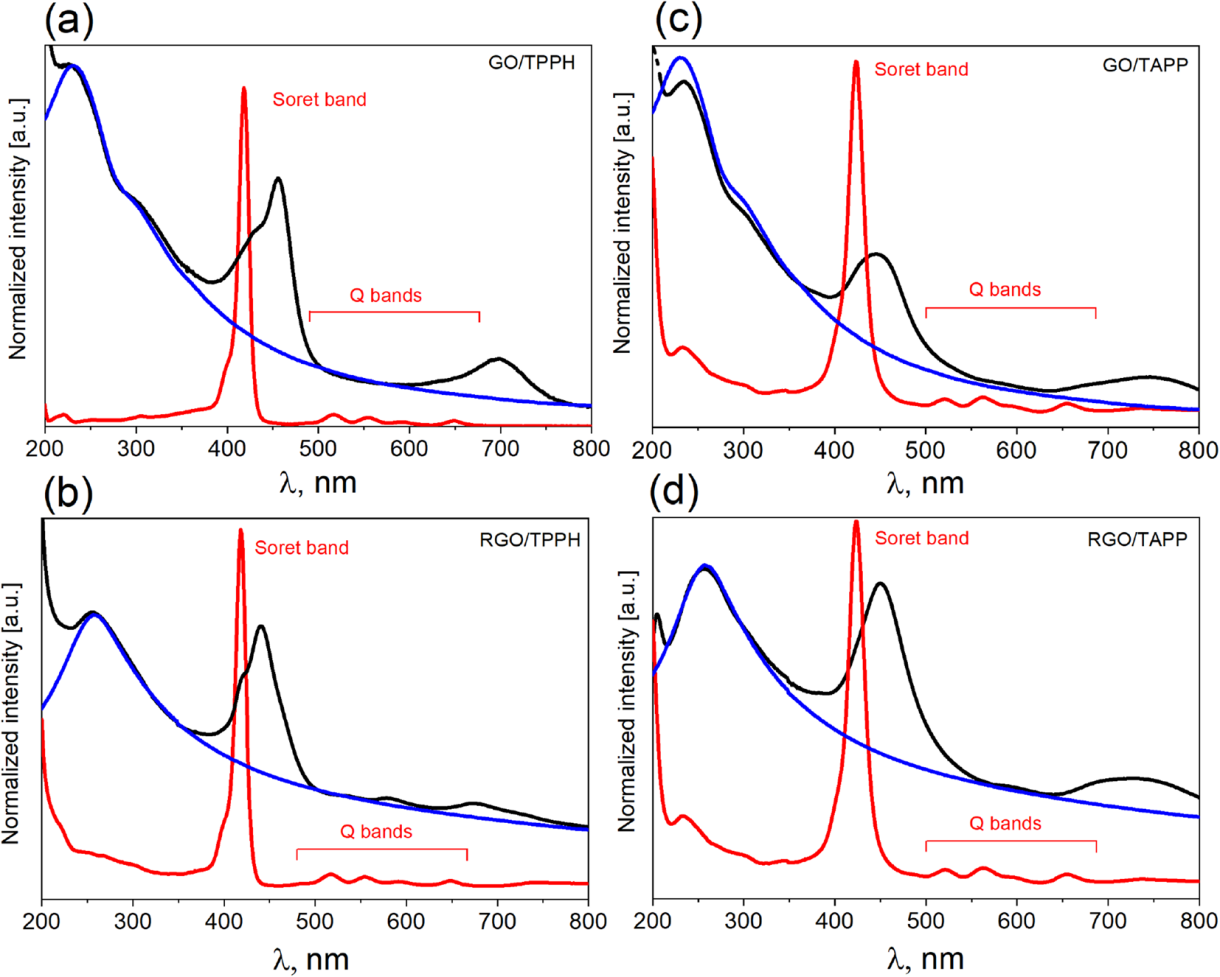
Experimental absorption spectra obtained for all nanohybrids studied (black lines): (**a**) GO/TPPH, (**b**) RGO/TPPH, (**c**) GO/TAPP, and (**d**) RGO/TAPP. In each case, the spectra for free TPPH and TAPP are shown in red while the spectra for free GO and RGO species are presented in blue. In the case of porphyrins, Soret and Q bands are marked.

UV–Vis absorption spectra for porphyrins and their nanohybrids with GO and RGO are depicted in Fig. 2. At a neutral pH both porphyrins exist in their neutral form. It is notable that the presence of graphene has a remarkable effect on the UV–Vis spectra of both porphyrins. Upon increasing GO or RGO concentrations we observed a disappearance of the porphyrins Soret bands and increase of intensity of the new Soret bands (SI Fig. S4 and S5). An isosbestic point was also observed in all cases confirming clear transformation of the free porphyrin into porphyrin adsorbed on the graphene type material (Table 1). The location and number of Q bands changed as well. Three of the four Q bands completely disappeared upon TPPH-GO nanohybrids formation, and a new broad band was observed at 699 nm. Disappearance of the Q-bands and formation of the broad band at 750 nm was also observed for both TAPP hybrids with both GO and RGO. However, for the TPPH-RGO the three Q-bands are present in the spectra but their position has changed (Table 1). Interestingly, the band at 681 nm has much lower intensity for TPPH-RGO than the band at 699 nm for TPPH-GO. The presence of this band may indicate a partial charge transfer from the TPPH to the GO sheet, resulting in the formation of positive charge, similarly to GO nanohybrid with closely-related ZnTPPH porphyrin^31^.

**Table 1:** Absorption properties of the free TPPH and TAPP and porphyrins adsorbed on GO and RGO.

Pophyrin	Free (nm)		GO (nm)			RGO (nm)			GO (mg)	RGO (mg)
	Soret band	Q bands	Soret band	Q bands	Isobestic point	Soret band	Q bands	Isobestic point		
TPPH	418	517, 555, 592, 650	452	699	428	442	537, 580, 680	429	0.033	0.19
TAPP	424	521, 563, 591, 654	456	750	437	451	750	439	0.085	0.42

The summary of the maximum amount of the TAPP and TPPH adsorbed per 1 mg of GO and RGO sheets is provided in the last two columns.

One can notice that the Soret band of the TPPH-GO and TPPH-RGO exhibits a significant red-shift of 34 nm and 24 nm, respectively. Comparable shifts of the Soret bands were observed in the case of TAPP-GO (32 nm) and TAPP-RGO (27 nm). Moreover, the Soret bands of the nanohybrids exhibit approximately two times lower extinction coefficients compared to free TPPH/TAPP molecules with a slight broadening of the band^1^.

The bathochromic shifts of the Soret band observed upon nanohybrid formation could be explained by a flattening of the porphyrin molecules. Our hypothesis about porphyrin flattening when adsorbed on the GO/RGO sheet is supported by the theoretical calculations (*vide infra*) that predict the dihedral angle between the phenyl and porphyrin plane decreases upon complexation with GO/RGO. We also note that absorption spectra of nanohybrids with RGO feature significant alternation in high-energy region (> 350 nm) as compared to the free RGO. This indicates strong electronic interaction and orbital mixing upon nanohybrid formation.

In order to compare the strength of the interaction of TPPH and TAPP molecules with two different graphene materials (GO an RGO) we investigated the increase of the absorbance of the Soret band (after subtracting the GO/RGO absorbance) of the porphyrins adsorbed on GO or RGO as a function of their concentration (SI Fig. S4c and S5c). The molar absorption coefficients of TPPH and TAPP adsorbed on GO and RGO are very similar. Thus, the increase in the absorbance is directly related to the concentration of the porphyrin molecules adsorbed on the GO/RGO sheets. For all four nanohybrids the Soret band absorbance increases linearly with the GO or RGO concentration. It was found that for TPPH and TAPP the slope of the linear regression of the absorbance change versus concentration of graphene material is five times higher for the RGO than for GO. Based on this analysis it can be concluded that porphyrins interact stronger with RGO. In line with this, we found that an order of magnitude more TPPH or TAPP can be absorbed onto 1 mg of RGO compared to GO sheets (see Table 1)^2^.

Batochromic shift of the Soret bands together with the decrease of the molar absorption coefficients upon addition of GO was previously reported by us for cationic porphyrins^47,48^. Interestingly, the spectral changes occurring upon addition of GO to the aqueous solutions of cationic porphyrins were more pronounced than for TAPP and TPPH since approximately ten times lower concentration of GO was required to achieve detectable spectral changes. Therefore, it is reasonable to conclude that the interaction of GO with neutral porphyrins TAPP and TPPH is weaker in comparison to positively charged porphyrins. It can be explained by the electrostatic attraction between cationic porphyrin and negatively charged GO that facilitate assembly of the naonhybrid material.

#### Emission

**Figure 3:**
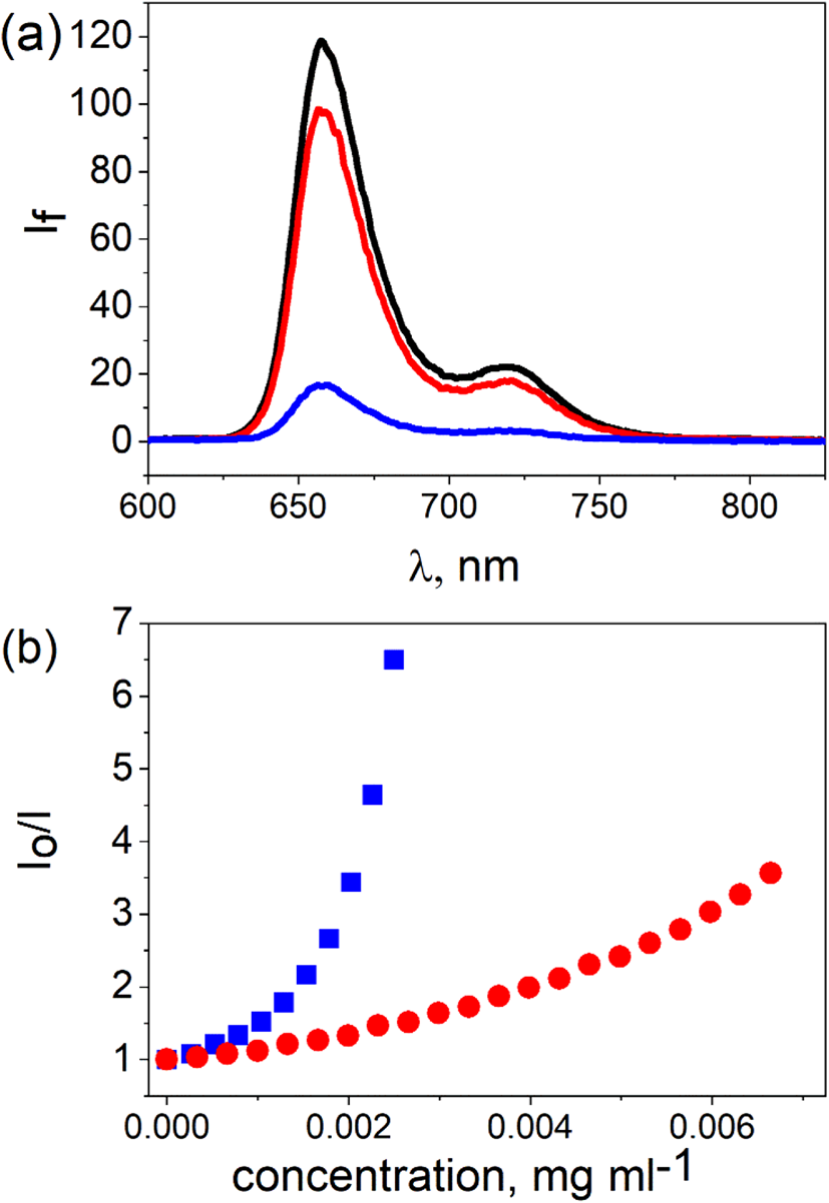
(**a**) Quenching of the fluorescence of $1.0\, \upmu \hbox {M}$ TPPH (black) recorded after the addition of $2.6 \times 10^{-3}$ mg ml$^{-1}$ of an aqueous suspension of GO (red) and RGO (blue). Spectra were corrected for the inner filter effect. (**b**) The relationship between fluorescence intensity $I_{0}/I$ ($I_{0}$ = Intensity without GO or RGO, *I* = Intensity after addition of GO or RGO) and GO (red) or RGO (blue) concentration.

**Figure 4:**
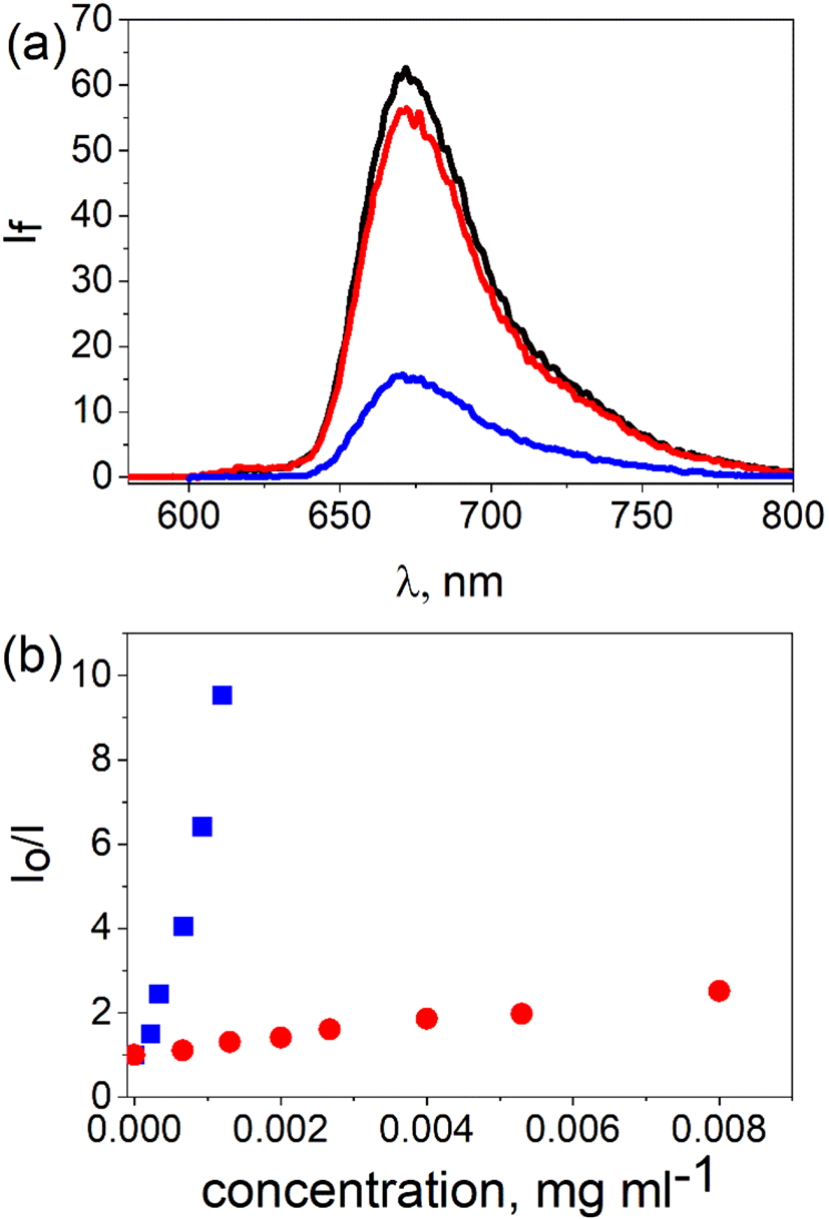
(**a**) Quenching of the fluorescence of $2.0 \,\upmu \hbox {M}$ TPPH (black) recorded after the addition of $6.9 \times 10^{-3}\,\hbox {mg ml}^{-1}$ of an aqueous suspension of GO (red) and RGO (blue). Spectra were corrected for the inner filter effect. (**b**) The relationship between fluorescence intensity $I_{0}/I$ ($I_{0}$ = Intensity without GO or RGO, *I* = Intensity after addition of GO or RGO) and GO (red) or RGO (blue) concentration.

The interaction of the excited states of the porphyrins with the GO and RGO sheets was investigated by the emission spectroscopy. It is worth mentioning that comparisons of the emission data require a matching of the absorbances at the excitation wavelength. In the current work, emission experiments were performed with the excitation at the isosbestic points which ensured constant absorbance (Table 1). In addition the emission data was corrected for the inner filter effect I and II. TPPH itself has, in EtOH–$\hbox {H}_{2}\hbox {O}$ (1:2 v/v), a broad emission comprising two unresolved Q(0,0) and Q(0,1) bands at ca. 657 and 719 nm, respectively (Fig. 3a). The emission spectra of TAPP has one broad band centered at 675 nm (Fig. 4a).

A decrease in the fluorescence intensities of both porphyrins is observed with increasing GO and RGO concentrations (see Figs 3b, 4b). In the latter case, the decrease of the fluorescence is more drastic. Emission quenching for neutral porphyrins TAPP and TPPH with graphene-based materials for which interaction is attributed mainly to $\pi$-$\pi$ stacking interactions was found to be less efficient in comparison to reported earlier fluorescence quenching of cationic porphyrins by GO^47,48^. More efficient emission quenching for cationic porphyrins was evidenced by ca. ten times higher slope of $I_{0}/I$ versus GO concentration in comparison to TAPP and TPPH. Quenching is often related to electron or energy transfer. However, steady state emission measurements alone in the presence of graphene do not provide enough information to determine the quenching mechanism. For this reason, complementary techniques, such as time-resolved emission, are needed to verify the mechanism of the emission quenching.

By applying the time-correlated single photon counting technique it was found that the emission decay profiles of TAPP and TPPH did not change upon addition of either GO or RGO (see SI Fig. S8 and S9). Based on the analysis of the steady-state and time-resolved emission data we thus conclude that the observed decrease of emission intensity in the presence of graphene materials is solely attributed to static quenching. Static quenching as the reason for the observed decrease of the porphyrins fluorescence in the presence of graphene-type materials has been reported previously by us for the related systems^31,47,48^.

Since all four investigated nanohybrids have a distinct ground state electronic structure as indicated by the change in their UV–Vis absorption spectra compared to the absorption spectrum of the unbound porphyrins (see Fig. 2), any emission from the complex should be red shifted compared to that of free TPPH and TAPP. However, upon addition of GO or RGO to the porphyrins solutions, no change in the shape as well as in the position of the peaks in the emission spectra was observed. Moreover, the fluorescence excitation spectra recorded for the porphyrins solutions after addition of graphene materials, matched the respective absorption spectrum of the free TPPH or TAPP (see SI Fig. S10 and S11). The results clearly demonstrate that the obtained nanohybrids are not an emissive material. Since fluorescence was not detected for any of the nanohybrids, a very fast deactivation process is evident, presumably electron transfer.

### Quantum chemical calculations

A series of quantum chemical calculations allowed us to gain detailed insights into the electronic structures of the obtained nanohybrids and their formation processes. In the first step we optimized the structures of isolated TAPP and TPPH molecules as well as the LK-type structural models of GO ($\hbox {C}_{59}\hbox {O}_{26}\hbox {H}_{26}$) and RGO ($\hbox {C}_{59}\hbox {O}_{17}\hbox {H}_{26}$). Analogous calculations were performed for the four nanohybrids. This allowed as to track back the structural changes that occur upon nanohybrid formation investigate key electronic factors that influence the interaction energies. Subsequently, the structures were subject to absorption spectra calculations as well as high-level ab initio computations of low-lying excited states. In this way we obtained state energy diagrams that are used to explain photochemical behavior of the examined systems.

#### Nanohybrid formation

**Figure 5:**
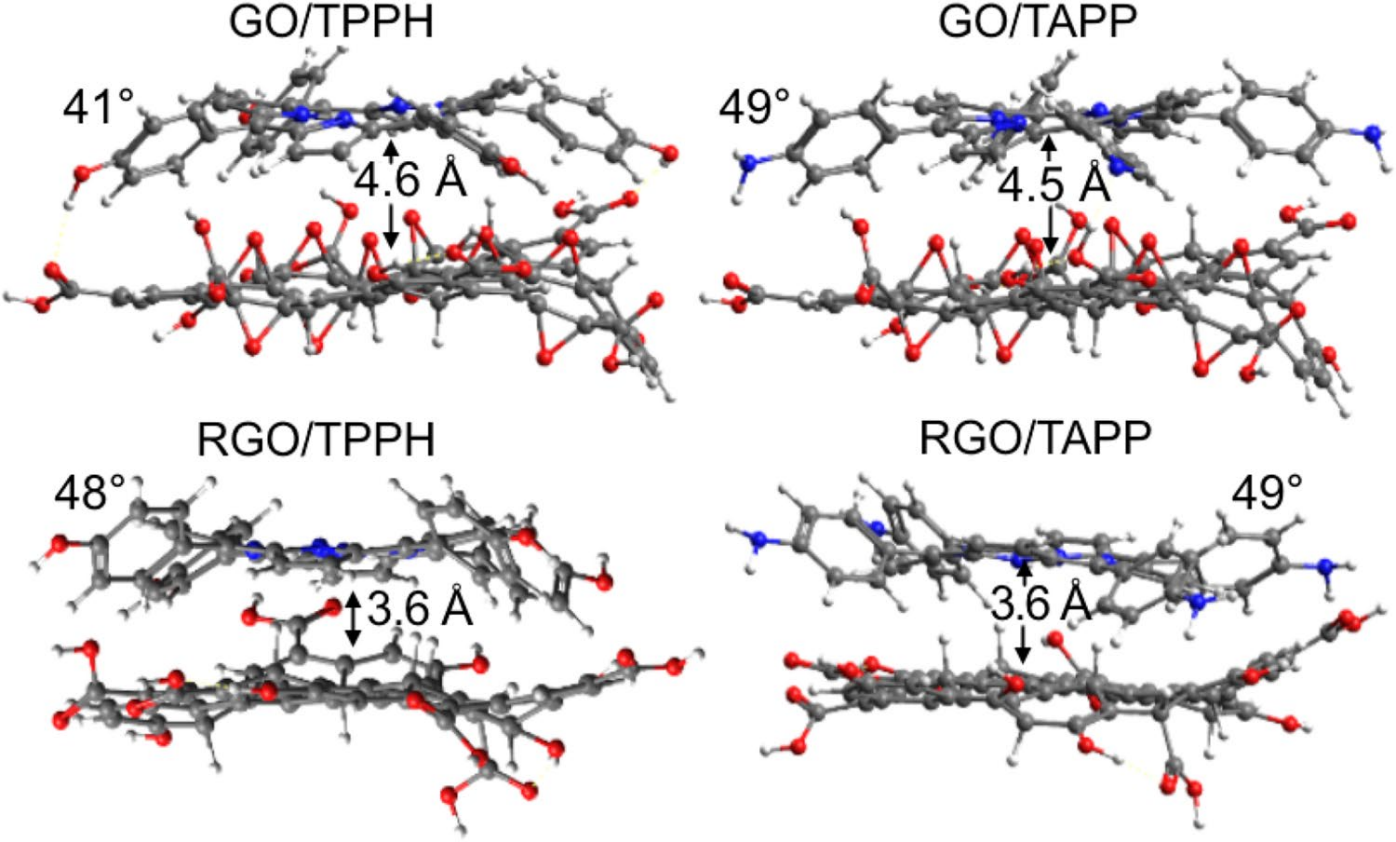
Geometries of the optimized nanohybrids. Highlighted are the distance between porphyrin and GO/RGO planes as well as dihedral angle that describes the porphyrins’ phenyl ring out-of-plane rotation.

Key geometric properties of the optimized structural models of the nanohybrids are shown in Fig. 5. The presence of GO or RGO influences both porphyrins in a similar way, i.e. the molecules become more planar as indicated by the decrease of the dihedral angle describing out-of-plane tilt of the phenyl rings. It is about $60^{\circ }$ for the isolated porphyrins while it ranges between $41^{\circ }$ and $49^{\circ }$ in the case of the nanohybrid. Interestingly, complexes that involve RGO model feature porphyrin rings on average 1 Å closer to the graphene plane than in the case of the GO species. Thus, we expect the former interaction to be more pronounced.

**Figure 6:**
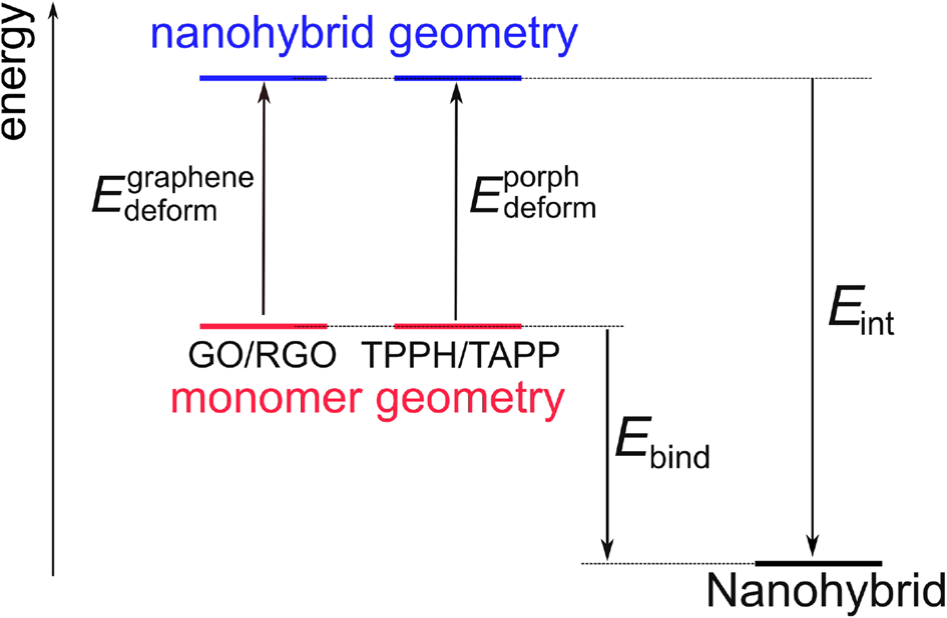
Binding energy ($E_{bind}$) decomposition scheme used to break down the energetic effect of the nanohybrid formation into three energetic components: geometric deformation of the substrates, $E_{deform}^{graphene}$ and $E_{deform}^{porph}$ as well as interaction energy of the deformed substrates ($E_{int}$).

According to the results presented in the Table 2 binding energies of both porphyrins to GO/RGO systems are strongly negative. Evidently, RGO tends to form more stable complexes complexes (see exact numbers in the Table 2). Further decomposition of the binding energies was performed into contributions that come from the deformation energies of the monomers ($E_{deform}$) and interaction between the deformed substrates ($E_{int}$) according to the scheme presented in the Fig. 6. Such decomposition is similar to activation-strain analysis of Bickelhaupt and co-workers^49–51^ and allows to distinguish between structural and electronic factors that contribute towards binding energy. We found that the RGO deformation energy upon nanohybrid formation is more than 5 kcal mol$^{-1}$ lower compared to GO. Moreover, the TAPP deformation energies were systematically smaller than corresponding values for TPPH. That translates into overall stronger affinity of TAPP to GO/RGO species compared to TPPH in agreement with experimental observations. The interaction energies are larger for RGO, presumably due to enhanced $\pi$–$\pi$ stacking interactions. In this case we also expect higher degree of orbital mixing that should manifest itself in the density of excited states.

**Table 2:** Binding energy ($E_{bind}$) released upon nanohybrid formation and its decomposition according to the scheme presented in Fig. 6.

Nanohybrid	$E_{bind}$	$E_{deform}^{graphene}$	$E_{deform}^{porph}$	$E_{int}$
TPPH-GO	− 21.2	14.9	13.9	− 50.0
TAPP-GO	− 24.4	18.4	7.3	− 50.1
TPPH-RGO	− 36.2	10.1	9.2	− 55.5
TAPP-RGO	− 36.7	8.5	5.9	− 51.1

All values are in $\hbox {kcal mol}^{-1}$.

#### Absorption spectra simulations

**Figure 7:**
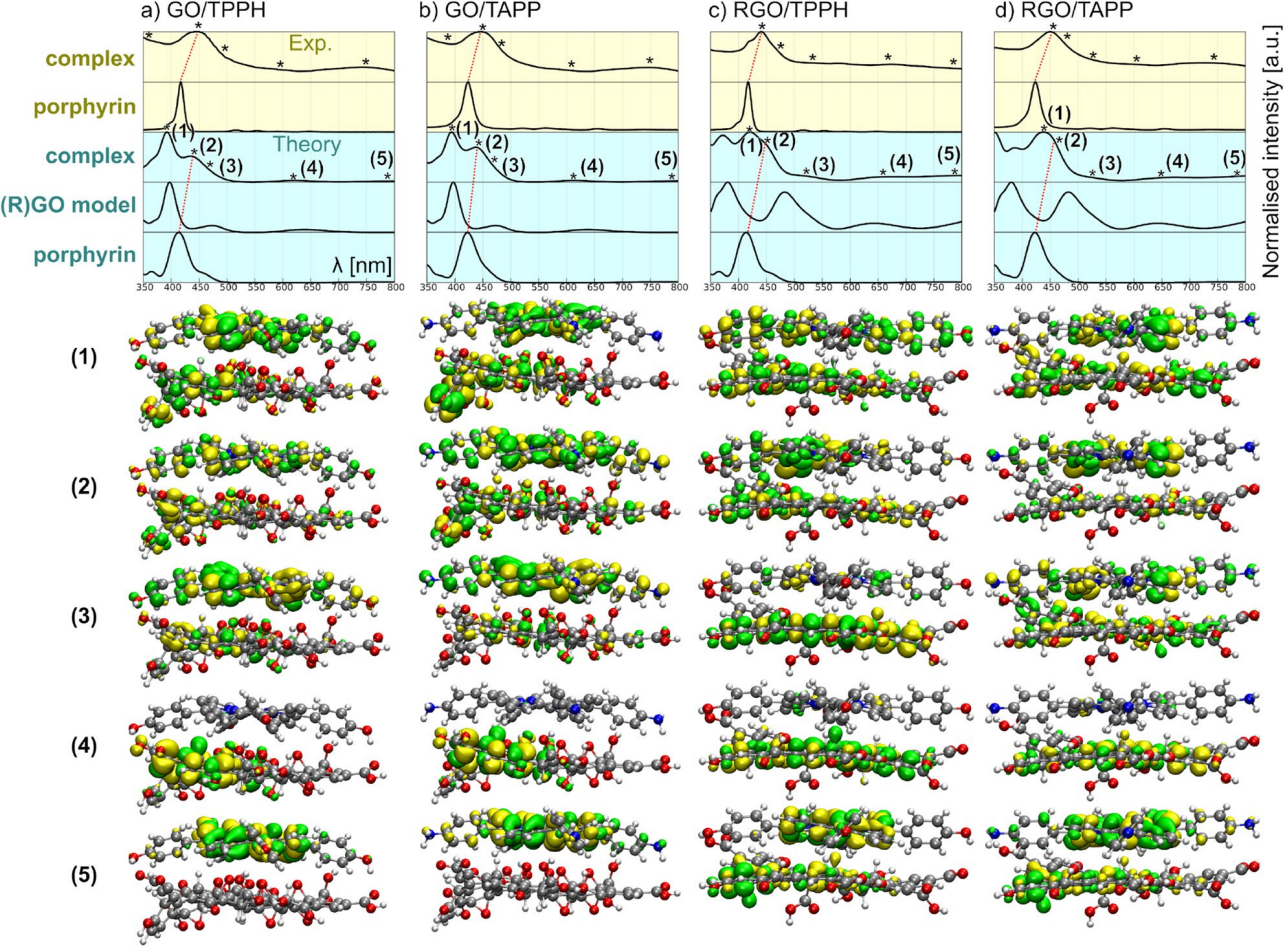
Top: comparison of experimental (yellow background) and computed (cyan background) absorption spectra. For each panel the spectra plotted are from the top: nanohybrid (exp), porphyrin (exp), nanohybrid (theory), graphene species (theory) and porphyrin (theory). Red dotted line guides identification of Soret-band shift in theory and experiment. Bottom: isosurface plots of transition densities for key electronic excitations (1)–(5) for all systems studied.

Figure 7 provides comparison of the experimental and computed UV–Vis absorption spectra. Experimental absorption spectra are relatively broad and feature a common maximum around 450 nm. Soret band shifts upon porphyrin adsorption on GO/RGO sheet are well reproduced in our calculations. We note that in the case of GO complexes, the final nanohybrid spectrum can be relatively easy decomposed into two contributions: from GO and TAPP/TPPH. This is not the case for RGO where the underlying RGO spectrum changes significantly upon complexation. The same was observed experimentally (*vide supra*).

For each computed spectrum we identified five key transitions (representatives of the most prominent bands) and traced their presence in experimental spectral curves. The computed transition densities for these transitions (1)–(5) are shown in the bottom of Fig. 7. For GO, the low-energy transitions (4) and (5) are GO and TAPP/TPPH-centered, respectively. In the case of RGO, it is apparent that Q-bands of the nanohybrids will have some contributions from RGO-centered excitations. When moving up in energy, charge-transfer excitations (CT), (3) are observed just before Soret band (2). The latter is strongly affected by the presence of graphene species. Finally, higher energy region (1) contains both CT and graphene-centered (not shown) excited states.

The comparison of experimental and computed UV–Vis spectra can also be used to distinguish the non-covalent and covalent functionalization of the graphene oxide. Many reports show that GO epoxy groups can, under certain circumstances, undergo nucleophilic attack that causes ring-opening and covalent linkage of the porphyrin to the GO support^52–57^. However, most of the reported reactions of the epoxy ring-opening require prolonged stirring or sonication, usually at elevated temperatures. In our case, the suspensions were mixed, followed by immediate spectroscopic measurement. However, if the covalent functionalization would take place in our set-up, it would lead to the porphyrin system being located perpendicularly to the GO plane. In this way, $\pi$–$\pi$ stacking would be disfavoured, and the shift of the Soret band should be minor. To test this hypothesis explicitly, we have carried out additional calculations on the nanohybrid where the TAPP molecule is indeed bound to a graphene oxide model with a covalent bond formed via ring-opening reaction. According to SI Fig. S12, such functionalization leaves the Soret band unshifted, confirming the non-covalent mode of interaction for the species studied.

#### Excited states from multireference computations

Further insights into the nature of low-energy excited states can be obtained with multireference ab initio calculations. Our state-averaged approach provides access to singlet and triplet states simultaneously. Moreover, the underlying CASSCF wave-function covers static (strong) correlation issues while DLNO-NEVPT2 corrects excitation energies for the missing dynamic correlation. Thus, the method can efficiently handle near-degeneracies within the active space.

**Figure 8:**
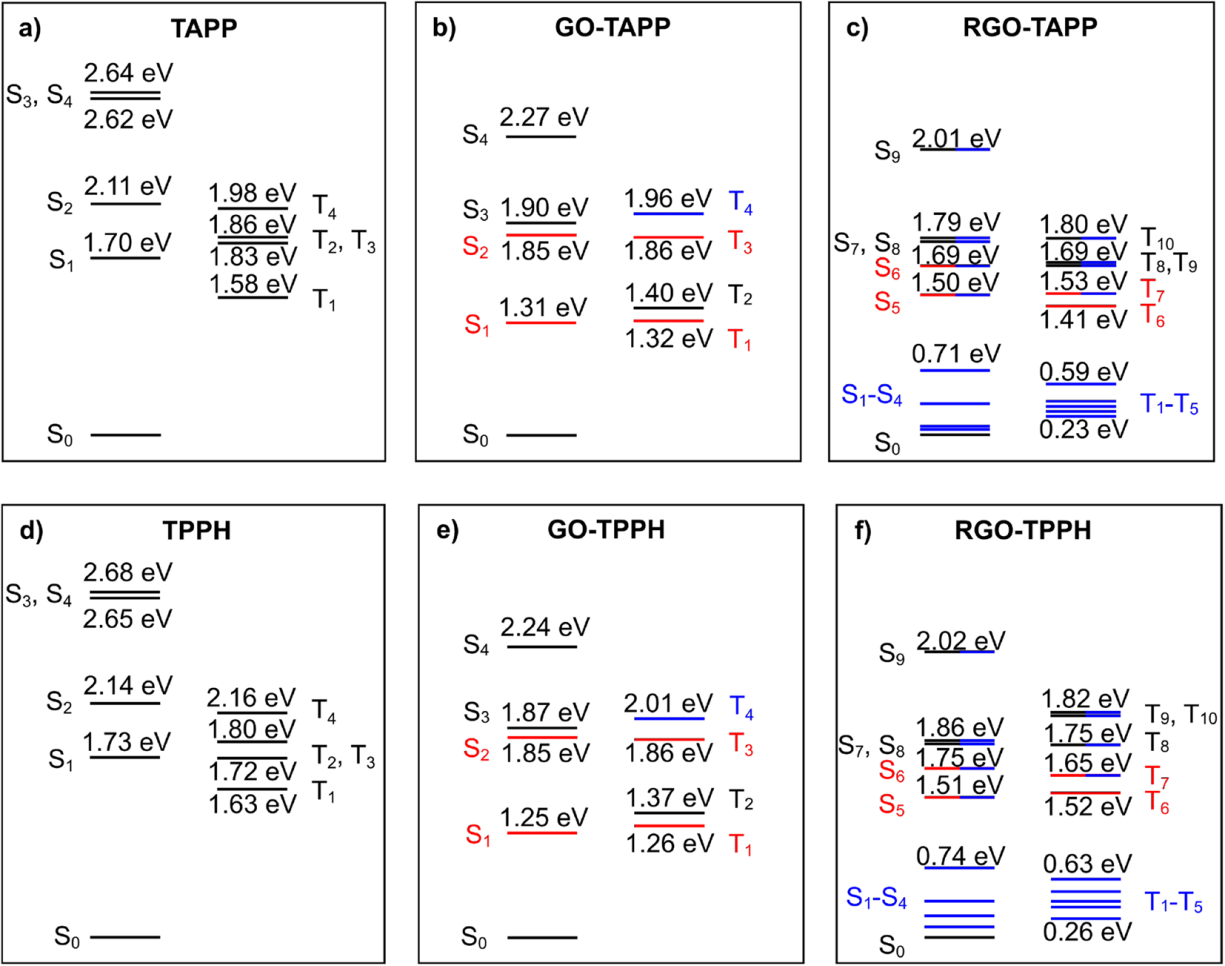
State-energy diagram for TAPP (**a**) and its nanohybrids with GO (**b**) and RGO (**c**) as well as analogous diagrams for TPPH species (**d**–**f**). State energies were computed at the DLPNO-NEVPT2 level. Excited states are classified as TAPP/TPPH-centered, GO/RGO-centered and charge-transfer and are marked in black, blue and red, respectively. Mixed states are denoted with two colors corresponding to dominant configurations.

The outcomes of CASSCF/DLPNO-NEVPT2 calculations are summarized in a form of state-energy diagrams shown in Fig. 8. For the free TAPP and TPPH, the common $\hbox {S}_{1}/\hbox {S}_{2}$ (and $\hbox {T}_{1}/\hbox {T}_{2}$) degeneracy is lifted by the presence of two hydrogen atoms (cf. with ZnTPPH molecule where the degeneracy is conserved^31^). The first singlet excited state of TAPP (1.70 eV) is found 0.03 eV lower in energy than in the case of TPPH, while the first triplet state is found in both cases around 1.6 eV.

Once the nanohybrids with GO are formed, the nature of the first excited singlet and triplet states changes from porphyrin-centered to charge transfer (CT). The low-lying $\hbox {S}_{1}$ CT states are found to be 1.31 and 1.25 eV above the ground state for GO/TAPP and GO/TPPH, respectively. However, in both cases the corresponding $\hbox {S}_{1} \leftarrow \hbox {S}_{0}$ transitions are only of very little intensity ($f_{osc} \approx 10^{-4}\,\hbox {a.u.}$) as compared to the $\hbox {S}_{2} \leftarrow \hbox {S}_{0}$ transitions ($f_{osc} \approx 0.03\,\hbox {a.u.}$). The latter transition is very close in energy to the excitation responsible for the observed Soret band so its detection is limited. The triplet states manifold is evidently brought down upon nanohybrid formation. It is worth mentioning that $\hbox {T}_{1}$ state is found to exhibit a CT character.

The nanohybrids that involve RGO model are more complex to analyze. We found high density of states close to the ground state. In fact 5 electronic states were found to be no more than 0.7 eV apart from each other. These are only RGO-centered excitations but this underlines the complex behavior of the RGO material. For both porphyrins, their complexes with RGO feature CT-capble excited states around 1.5 eV. However, in contrast to GO these states have strong multireference character, i.e. CT configuration state functions (CSFs) are accompanied with CSFs that involve only RGO-centered orbitals. This once again points to strong orbital interaction between porphyrins and RGO species.

## Conclusions

Presented study has two major outcomes. On one hand side, by carrying out careful spectroscopic analysis, we found the two examined porphyrins form stable nanohybrids with GO/RGO support. Here, for the first time we showed that both porphyrins exhibit higher affinity to RGO. On the other hand, we demonstrated that LK-type models allow for robust representation of the GO/RGO surfaces in quantum chemical calculations. The models pave the way for applications of high-level ab initio methods to solid-state problems owing the local nature of adsorbate-GO/RGO interaction.

The spectra simulated with efficient $\hbox {sTDA}/\omega \hbox {B97X}$ protocol presented in the manuscript were shown to provide very good agreement with the experimental curve shapes. Theoretically predicted shifts upon nanohybrid formation agree very well with those derived experimentally in the present study. Low-lying excited states were analyzed with the state-of-the-art DLPNO-NEVPT2 approach.

The origin of strong porphyrin-RGO interactions was traced back to the small deformation energy of the RGO upon nanohybrid formation as well as to more extended availability for $\pi$–$\pi$ stacking interactions. The latter is particularly important as the porphyrins get more planar in the complex as compared to free molecules. We have also demonstrated that interaction with the RGO is much more complex than in the case of the GO due to strong orbital mixing. Moreover, experimentally accessible excited states were found to feature strong CT character that leads to fast deactivation process (electron transfer) and makes the materials non-emissive.

Taking into account the variety of the possible porphyrin/graphene materials the development of theoretical methods that could properly predict spectroscopic properties of those materials can serve as a guidance for fabrication of such materials with desired properties.

## Methods

### Experimental details

#### Materials

5,10,15,20-tetrakis(4-aminophenyl) porphyrin (TAPP) and 5,10,15,20-tetrakis(4-hydroxyphenyl) porphyrin (TPPH) were purchased from Porphyrin Systems, graphite powder was purchased from Acros Organics and ascorbic acid was purchased from Sigma Aldrich. Ethanol (HPLC grade) was bought from J.T. Baker. Solutions were prepared with millipore distilled water ($18\,\hbox {M}\Omega \,\hbox {cm}$). The purchased chemicals were used without further purification.

#### Preparation of GO and RGO

GO was obtained *via* a modified Hummers’ method. Concentrated $\hbox {H}_{2}\hbox {SO}_{4}$ (230 mL) was mixed with graphite powder (10 g) at a temperature below $10\,^{\circ }\hbox {C}$. Next, $\hbox {NaNO}_{3}$ (4.7 g) was added with constant cooling of the reactor in an ice bath. After 15 min $\hbox {KMnO}_{4}$ (30.0 g) was slowly, gradually added to the mixture so that the temperature did not exceeded $10\,^{\circ }\hbox {C}$. Then the mixture was slowly heated to $30\,^{\circ }\hbox {C}$ and stirred for two hours. Subsequently, 100 mL of deionized water was added, and the temperature was raised to $100\,^{\circ }\hbox {C}$. The reaction mixture was stirred for 20 min at ca. $100\,^{\circ }\hbox {C}$. Afterwards, 10 mL of hydrogen peroxide (30% solution) was slowly added.The obtained dark yellow suspension was sonicated for 1 h using an ultrasonic bath (Bandelin Sonorex RK 106), after which the slurry was filtrated and thoroughly washed with deionized water until the pH of the filtrate reached 6.5.

RGO was obtained by a chemical reduction of GO ($0.1\,\hbox {mg}\,\hbox {mL}^{-1}$ water) using an excess of ascorbic acid (0.1 M) at pH 10 adjusted by NaOH, according to an earlier described procedure^58,59^. The reaction mixture was stirred and kept of $70\,^{\circ }\hbox {C}$ for 5 h until the brown suspension turned black. Afterwards the obtained suspension was centrifuged at 12,000 rpm (14986 rcf) for 30 min and washed with water several times in order to remove any excess of ascorbic acid. The wet solid was transferred into a Petri dish and dried in an oven for 24 h at $60\,^{\circ }\hbox {C}$. Under a mild sonication, the obtained RGO could be re-dispersed in water.

The GO and RGO materials were characterized by absorption spectroscopy, thermogravimetric analysis, photoelectron spectroscopy (XPS) and FTIR as described previously^59^.

#### Spectroscopic measurements

UV–Vis absorption spectra were recorded using a dual-beam spectrometer Cary 100 (Agilent) UV–Vis scanning from 200 to 800 nm with 1 nm increments. Quartz cuvettes with 10 mm optical path lengths were used.The fluorescence spectra for solutions with 0.1 and lower absorbance at the excitation wavelength were collected on LS 50B spectrofluorometer (Perkin Elmer). The samples were excited at the isosbestic point obtained during UV–Vis titration experiments with GO and RGO. Emission lifetimes were measured using a FluoTime300 fluorescence spectrometer (PicoQuant) operating in the time-correlated single photon counting mode (TCSPC). A light-scattering Ludox solution (colloidal silica) was used to obtain the instrumental response function (prompt). The emission decay lifetimes were measured following excitation with 440 nm or 405 nm photodiodes. Emission data was corrected for inner filter effect I and II^60^. All spectroscopic measurements were performed in EtOH-$\hbox {H}_{2}\hbox {O}$ (1:2 v/v) mixtures at neutral pH.

### Computational details

#### GO and RGO structural models

The chosen molecular structure of the graphene oxide (GO) model $\hbox {C}_{59}\hbox {O}_{26}\hbox {H}_{26}$ essentially represents the experimentally-derived model of Lerf et al.^44^ We consider a model system with a C/O atomic ratio of 2.3, that is close to the one reported for GO used in the experimental part of work^59^.

Similarly to GO, RGO is a finite system. The available structural information does not provide a clear understanding of the local microscopic structure. TEM data indicate the presence of quasi-amorphous $sp^{2}$-bonded areas^61^, this is in good agreement with XPS data that shows decreased content of $sp^{3}$-hybridized carbon atoms along with decreased content of epoxy and carbonyl groups in comparison to GO^62^. The RGO’s C/O ratio is ca. 4 which indicates that large amount of the oxygen-containing groups are successfully removed from GO upon reduction^59^. Therefore, in our calculations, we have initially considered three RGO models derived from our GO structure:

- $\hbox {C}_{59}\hbox {O}_{17}\hbox {H}_{26}$ model with epoxy groups removed,
- $\hbox {C}_{53}\hbox {H}_{28}$ defective graphene structure with terminal hydrogen atoms (other hydrogen atoms introduce simple defects in otherwise perfect $\pi$-conjugation),
- $\hbox {C}_{53}\hbox {H}_{18}$ flat graphene-like structure with terminal hydrogen atoms.

SI Fig. S1 provides comparison of the UV–Vis absorption spectra of the three RGO models (a)–(c) with the experimental absorption spectrum. Structures (a) and (c) were chosen on a basis of good qualitative agreement with the experimental data. The best match between experimental and computed UV–Vis absorption spectra was found to be for the model (a). Thus, presented calculations were based on RGO model (a) [for completeness, see Fig. S2 for absorption spectra of TPPH/RGO and TAPP/RGO nanohybrids obtained by applying different RGO, i.e. models (b) and (c)].

The GO and RGO models that have been used throughout the work are shown in Fig. 1c,d, respectively.

#### Geometry optimizations

BP86 functional^63^ supplemented with D3BJ dispersion correction^64,65^ was chosen as an optimal compromise between cost and accuracy. Geometrical counterpoise correction (gCP)^66^ was applied to minimize intra- and intermolecular basis set superposition error. Obtained structures were subject to numerical second derivative calculations and were confirmed to possess only positive normal modes.

#### Binding and interaction energies

Binding energies ($E_{bind}$) for TPPH/TAPP and GO/RGO molecules in the nanohybrids were computed according to following expression:

1 $$\begin{aligned} E_{bind} = E_{nanohybrid} - E_{TPPH/TAPP} - E_{GO/RGO} \end{aligned}$$

where the energies of the monomers at their optimal geometries were subtracted from the energy of the optimized nanohybrid. The interaction energy ($E_{int}$) was defined within the energy decomposition scheme (see Fig. 6). The ($E_{TPPH/TAPP} + E_{GO/RGO}$) contribution is calculated with the monomers’ geometries as found in the nanohybrid.

All single-point energies were computed with $\omega$B97X functional^67^ along with D3BJ and gCP corrections for the BP86+D3+gCP geometries. Zero-point energies calculated with the latter method were included as well.

#### Absorption spectra calculations

Spectra calculations were performed with two approximate time-dependent DFT methods: simplified time-dependent DFT (sTD-DFT)^68^ and simplified Tamm–Dancoff approach (sTDA)^69^. All states up to 10 eV were considered, resulting in more than 3000 states accounted for in each calculation. Hybrid B3LYP^63,70^ and range-separated $\omega$B97X functionals were tested. SI Fig. S3 provides summary of the benchmarks performed.

Interestingly, the sTDA was found to reproduce the best experimental curve shapes in our study. In the original sTDA work^69^ the author shows that by requiring Coulomb (*ii*|*aa*) terms in sTDA to approach $\frac{1}{R}$ behavior one may to some degree remedy the well-known problem of the TD-DFT methods with charge-transfer (CT) states. Thus, in combination with range-separated functional that provides better one-electron basis (Kohn-Sham eigenvalues), sTDA may thus provide reasonable description of the CT states. Therefore, our final setup combines sTDA approach and $\omega$B97X functional. The spectra were simulated by applying Lorentzian broadening with a broadening parameter of $2000\,\hbox {cm}^{-1}$. In addition, all of the computed spectra were uniformly red-shifted by $950\,\hbox {cm}^{-1}$ to visually match with the experimental data. The assignment of bands was carried out using transition density plots and by analyzing one-electron excitation contributions (see SI Fig. S13 and Table S1).

#### Multireference calculations

Starting vectors for complete active space self-consistent field (CASSCF)^71^ calculations were obtained from MP2 natural orbitals. Strongly occupied orbitals with occupation numbers smaller than 1.90 and weakly occupied orbitals with occupation numbers of more than 0.10 were selected to enter the active space in subsequent CASSCF iterations. In the case of free porphyrins, the procedure provided active space consisting of four essential Gouterman’s orbitals^72,73^ along with four electrons. This space is abbreviated as CAS(4,4). The complexes with GO and RGO were treated with spaces CAS(8,7) and CAS(8,8) that contain in addition to porphyrin-centered Gouterman’s orbitals also contain the orbitals of the graphene species (strongly and weakly occupied). Isosurface plots of all active space orbitals are provided in the SI.

The CASSCF wave-function was optimized in a state-averaged way. For TAPP/TPPH and their nanohybrids with GO the averaging was over 5 singlet and 4 triplet states. In the case of RGO the number of states was increased to 10 singlets and 10 triplets due to high density of states in the relevant energy window.

To account for dynamic correlation outside the active space, excitation energies were calculated with the n-electron valence state perturbation theory at the second order (NEVPT2)^74^. Due to the size of the systems under study, we employed the domain based local pair natural orbital approximation with the default settings (DLPNO-NEVPT2)^75^. The calculations were performed using compact def2-SVP basis set^76^. Test calculations for TPPH shown that an adapted set-up provides excitation energies within 0.02 eV error with respect to much more demanding canonical NEVPT2 computations that use def2-TZVP basis^77^.

#### Common techniques and software

If not stated otherwise, def2-TZVP orbital basis set^77^ was used throughout the study. Resolution-of-the-identity^78^ along with corresponding auxiliary basis set^79^ was employed in Coulomb integrals evaluation. Exchange integrals were evaluated seminumerically^80^. Geometry optimizations, interaction energies and multireference calculations were performed with ORCA 4.2.0 package^81^. sTDA and sTD-DFT computations were carried out with standalone stda 1.6.1.1 code obtained from author’s github repository^82^ and interfaced with ORCA and Turbomole 7.4 software^83^. The former was used for spectra generation while the latter allowed us to compute transition densities using *escf* module^84^ provided with sTDA-generated *ciss_a* file that contains excited state vectors in Turbomole’s format. Isosurfaces of transition densities ($\pm 0.001\,\hbox {a.u.}$) and molecular orbitals ($\pm 0.03\,\hbox {a.u.}$) were plotted using VMD 1.9.3 program^85^.

## Supplementary information

Supplementary Informations.

## Footnotes

1 The molar absorption coefficients for free TPPH and TAPP were determined by measuring the UV–Vis spectra of a solution of known concentration. The spectrum of the TPPH or TAPP adsorbed on the GO or RGO (SI Fig. S4d and S5d) was obtained based on the experiment described in SI Fig. S5a-b. By subtracting the spectrum of GO or RGO (obtained at the end of parallel titration of the blank sample in the same solvent) from that of the porphyrin/GO and porphyrin/RGO at the end of the titration of the porphyrin solution, a spectrum of the porphyrin adsorbed on the GO or RGO surface was obtained. In addition, an assumption that all TPPH and TAPP molecules were adsorbed on GO and RGO was made.

2 The estimations are based on the absorption spectra of GO and RGO that were recorded with the addition of varying amounts of concentrated TPPH or TAPP solution (see SI Fig. S6 and S7). With an increasing concentration of the porphyrins, we observed an increased contribution of the Soret band attributed to the free porphyrin.
