# Supplementary material for: Lerf–Klinowski-type models of graphene oxide and reduced graphene oxide are robust in analyzing non-covalent functionalization with porphyrins
Source: Sci Rep. 2021 Apr 12;11:7977. doi: 10.1038/s41598-021-86880-1 (PMC8041773; doi:10.1038/s41598-021-86880-1)
Supplement: Supplementary file 1 — Supplementary Informations. [file 41598_2021_86880_MOESM1_ESM.pdf]

Supplementary Information

Lerf-Klinowski-type models of graphene oxide and reduced graphene oxide are robust in analyzing non-covalent functionalization with porphyrins.

Alexandra Siklitskaya,[a] Ewelina Gacka,[b,c] Daria Larowska,[b] Marta Mazurkiewicz-Pawlicka,[d] Artur Malolepszy,[d] Leszek Stobiński,[d,e] Bronislaw Marciniak,[b,c] Anna Lewandowska-Andralojc,[b,c*] Adam Kubas,[a*]

[a]Institute of Physical Chemistry, Polish Academy of Sciences, Kasprzaka 44/52, 01-224 Warsaw, Poland, akubas@ichf.edu.pl

[b]Faculty of Chemistry, Adam Mickiewicz University, Uniwersytetu Poznanskiego 8, 61-614, Poznan, Poland, alewand@amu.edu.pl

[c]Center for Advanced Technology, Adam Mickiewicz University, Uniwersytetu Poznanskiego 10, 61-614 Poznan, Poland

[d]Faculty of Chemical and Process Engineering, Warsaw University of Technology, Warynskiego 1, 00-645 Warsaw, Poland

[e]*NANOMATERIALS Leszek Stobinski (www.nanomaterials.pl), Poland*

1. RGO model selection

Three models of graphene oxide were considered:

(a) $C_{59}O_{17}H_{26}$ model with epoxy groups removed,

(b) $C_{53}H_{28}$ defective graphene structure with hydrogen atoms used to terminate edges (other hydrogen atoms introduce simple defects in otherwise perfect π-conjugation);

(c) $C_{53}H_{18}$ flat graphene-like structure terminated with hydrogen atoms at the edges.

Figure S1 provides comparison of the sTDA computed spectra for these systems. High-intensity band between 600 and 700 nm excludes model (b). Model (a) has maximum at ~250 nm and an intense satelite ~350 nm. The latter disappears when going to model (c) along with maximum peak shift below 200 nm. In the experiment a broad tail above 300 nm is observed. Thus, we conclude that model (a) is best representation of the experimental species.

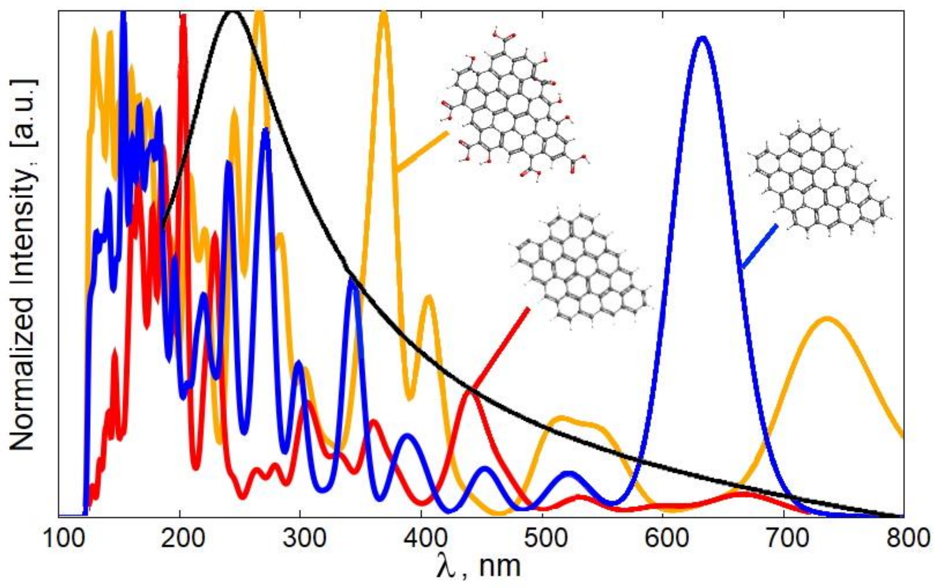

Fig. S1. Normalized and unshifted absorption spectra of three different reduced graphene models: (a) deepoxy (orange line), (b) defective graphene (blue line) and (c) simple graphene (red line).

For completeness, in Fig. S2 we provide complete comparison of experimental absorption spectra of RGO-TAPP/TPPH nanohybrids with those obtained with models (b) and (c) in analogy to main text.

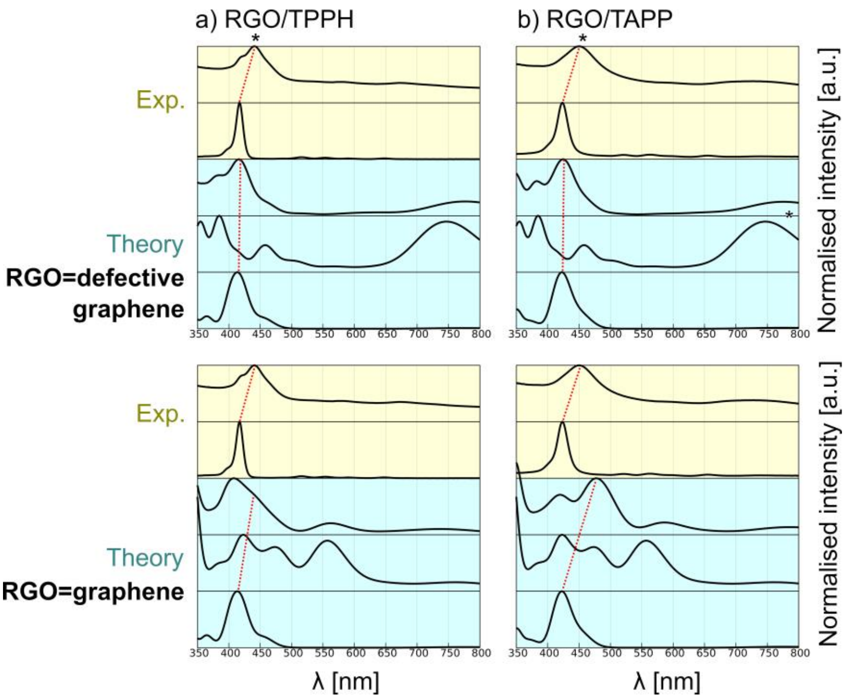

Fig. S2. Comparison of computed (cyan background) and experimental (yellow background) absorption spectra. For each panel the spectra plotted are from the top: nanohybrid (exp), porphyrin (exp), nanohybrid (theory), graphene species (theory) and porphyrin (theory). Red dotted line guides identification of Soret-band shift in theory and experiment.

2. Benchmark of the computational methodology for the absorption spectra calculation.

Figure S2 presents the comparison of spectra obtained with the sTD-DFT and sTDA approximations with two different exchange-correlation functionals (B3LYP and ωB97X) for the TPPH porphyrin. Both Soret and Q bands and reproduced best by combination of the sTDA/ωB97X methodology.

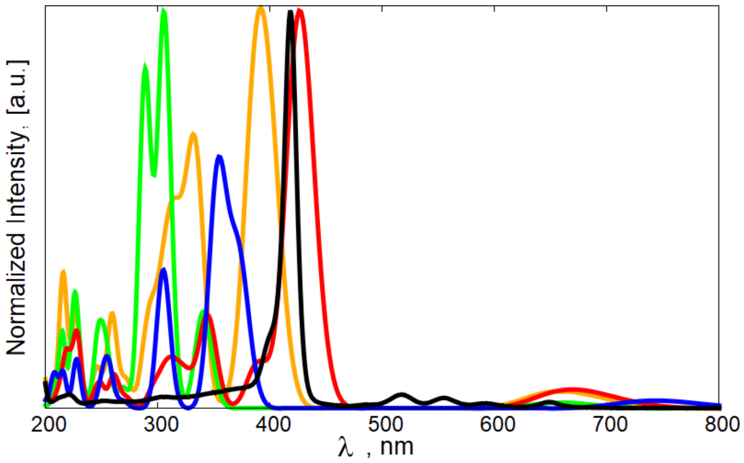

Fig. S3. Comparison of the experimental absorption spectra of the TPPH porphyrin (black line) with spectra computed with four setups: sTD-DFT/wB97X (blue), sTD-DFT/B3LYP (green), sTDA/wB97X (red) and sTDA/B3LYP (orange).

3. CASSCF active space orbitals plotted with ±0.03 a.u. isosurface.

TAPP: CAS(4,4)

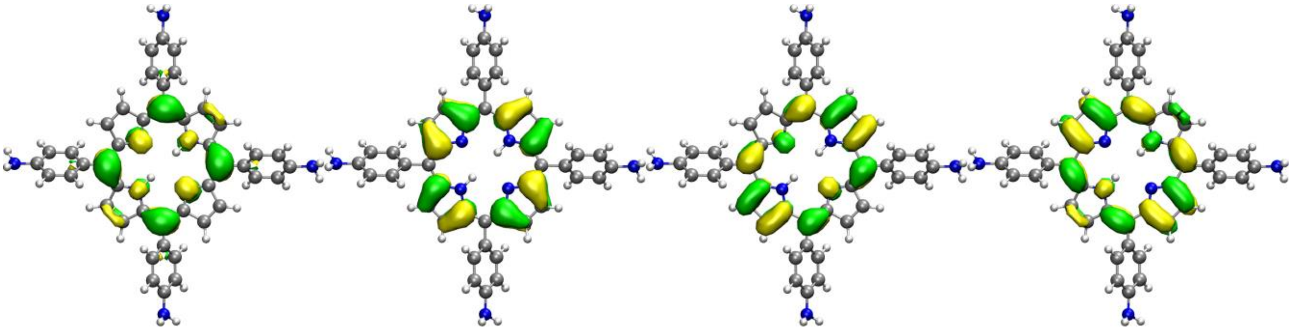

TPPH: CAS(4,4)

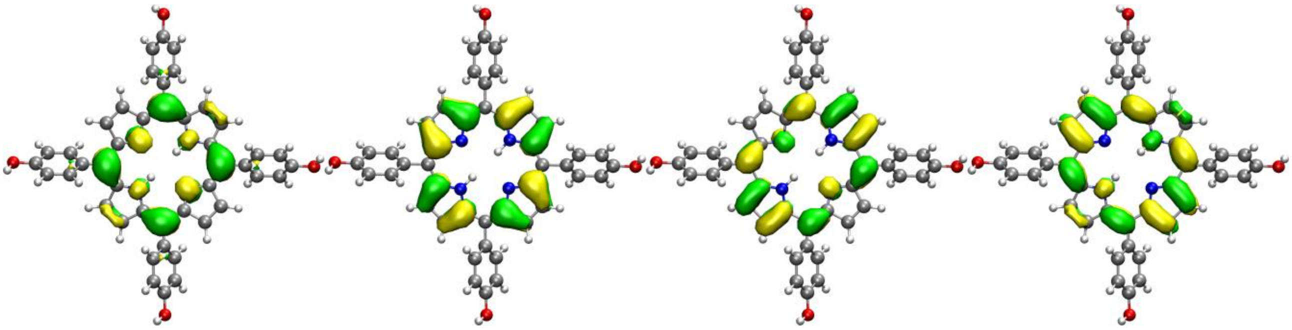

GO-TAPP: CAS(8,7)

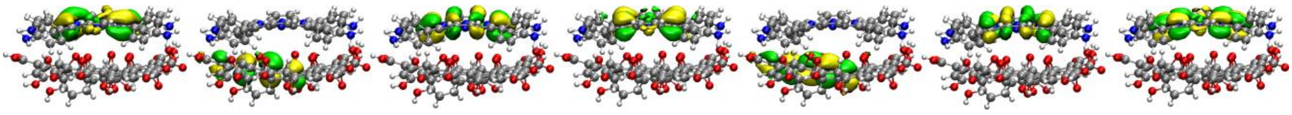

GO-TPPH: CAS(8,7)

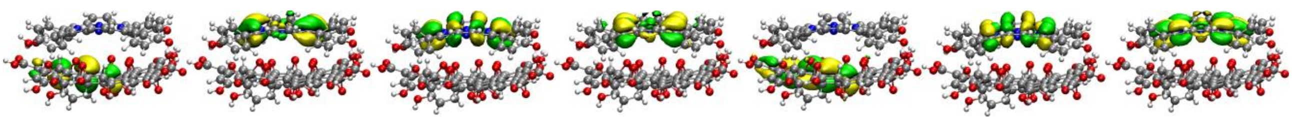

RGO-TAPP: CAS(8,8)

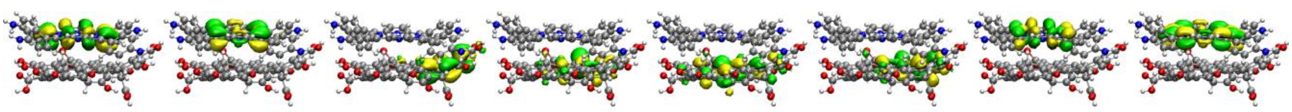

RGO-TPPH: CAS(8,8)

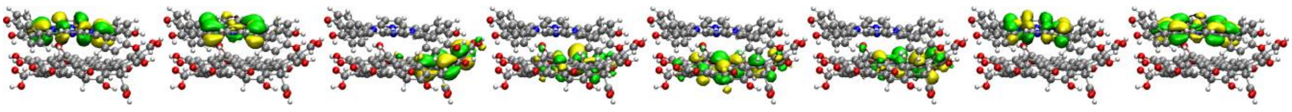

4. UV-vis spectra for a series of TPPH and TAPP suspensions measured for a constant porphyrin concentration and for varying GO or RGO concentration.

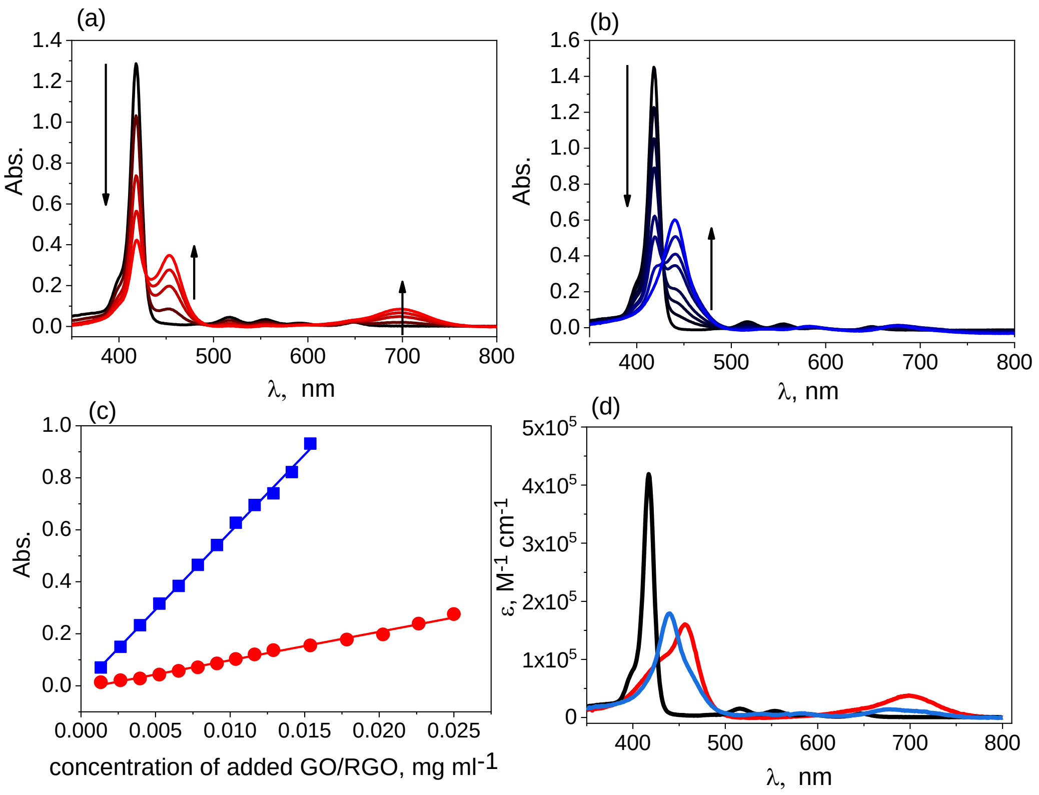

Figure S4. Absorption spectra recorded during the process of titration of 3 ml of 3.0 μM EtOH-H_2O (1:2 v/v) solution of TPPH with 0.4 mg ml^{-1} of a) GO dispersion (0-0.025 mg ml^{-1}), b) RGO (0-0.015 mg ml^{-1}), c) dependence of the absorbance at 452 nm (red) and 440 nm (blue) of TPPH as a function of the GO (red) and RGO (blue) concentration used for titration. d) absorption spectra of the free TPPH (black), TPPH adsorbed on GO (red), TPPH adsorbed on RGO (blue).

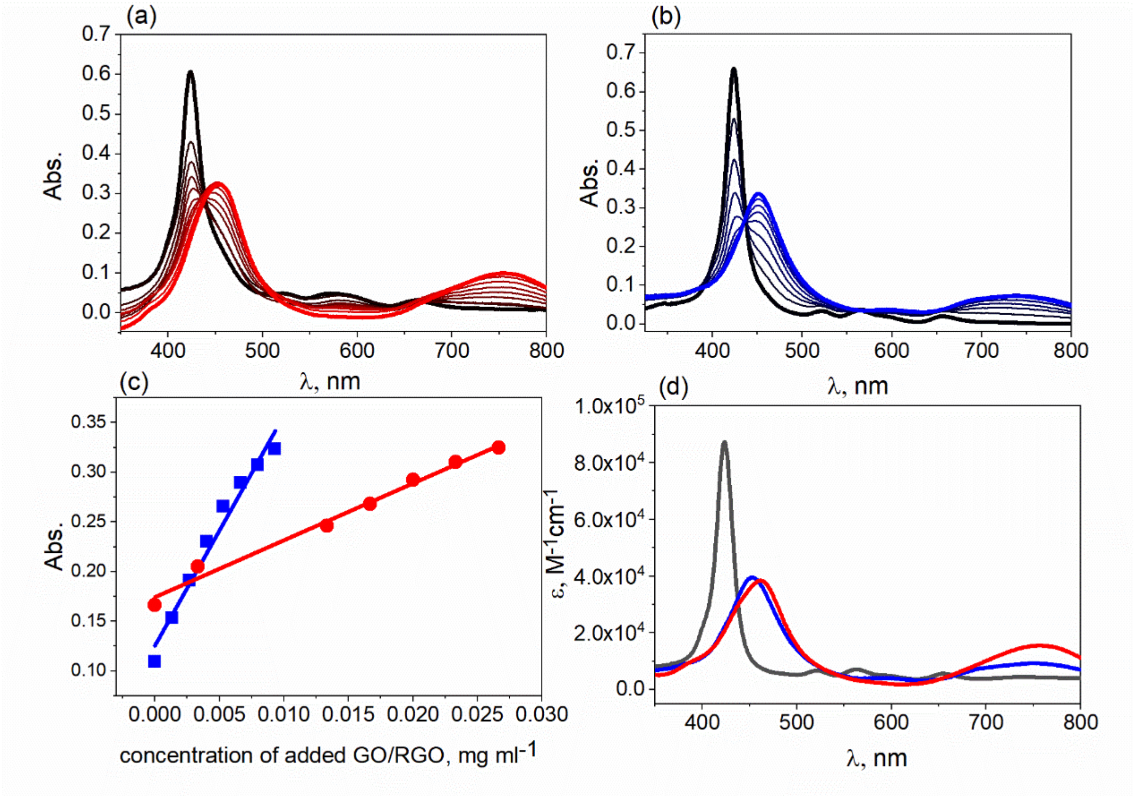

Figure S5. Absorption spectra recorded during the process of titration of 3 ml of 7.0 μM EtOH-H_2O (1:2 v/v) solution of TAPP with 1 mg ml^{-1} of A) GO dispersion (0-0.033 mg ml^{-1}), B) RGO (0-0.012 mg ml^{-1}), C) dependence of the absorbance at 456 nm (red) and 451 nm (blue) of TAPP as a function of the GO (red) and RGO (blue) concentration used for titration. D) absorption spectra of the free TAPP (black), TAPP adsorbed on GO (red), TAPP adsorbed on RGO (blue).

5. Absorption spectra of GO and RGO with the addition of varying amounts of concentrated TPPH or TAPP solutions

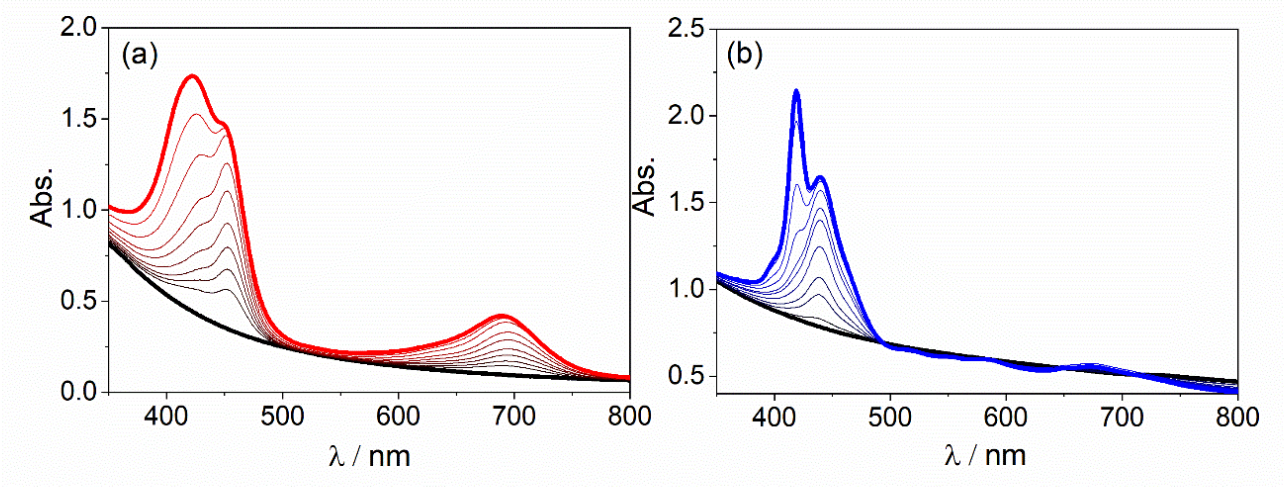

Figure S6. Absorption spectra recorded during addition of EtOH-H_2O (1:2 v/v) solution of TPPH to a) 0.1 mg ml^{-1} GO in H_2O (3 ml) b) 0.04 mg ml^{-1} RGO in H_2O (3 ml).

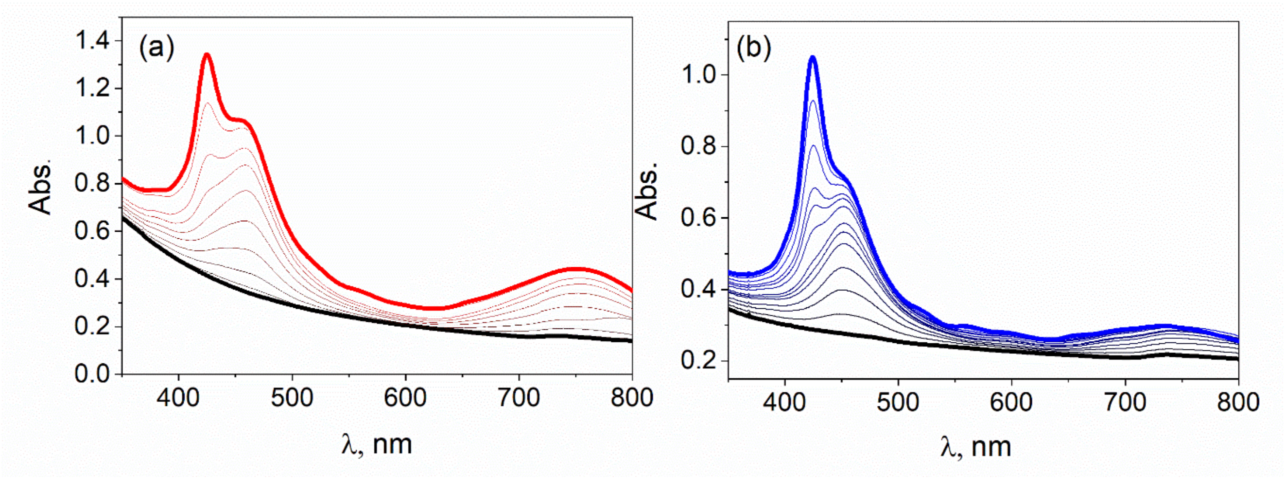

Figure S7. Absorption spectra recorded during addition of EtOH-H_2O (1:2 v/v) solution of TAPP to a) 0.1 mg ml^{-1} GO in H_2O (3 ml) b) 0.013 mg ml^{-1} RGO in H_2O (3 ml).

6. Time-correlated single photon counting measurements

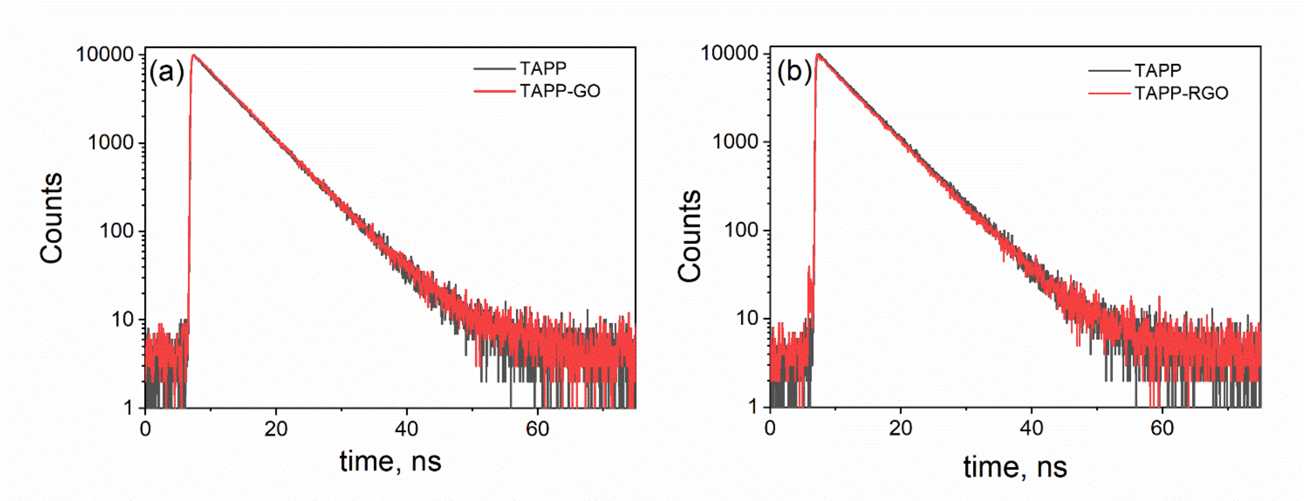

Figure S8. Decay of (a) TAPP fluorescence recorded in the absence (black) and presence (red) of GO (0.033 mg ml^{-1}); λ_{ex}= 440 nm, λ_{em}= 672 nm, (b) TAPP fluorescence recorded in the absence (black) and presence (red) of RGO (0.034 mg ml^{-1}); λ_{ex}= 440 nm, λ_{em}= 672 nm.

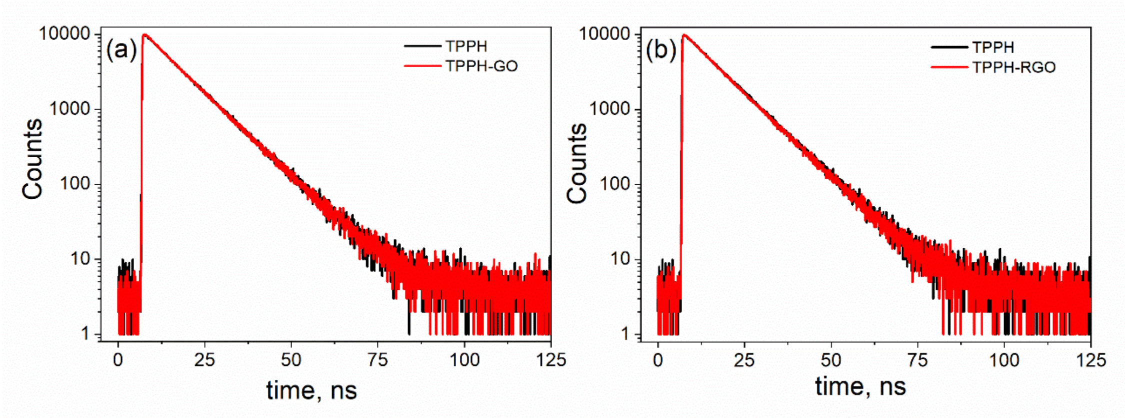

Figure S9. Decay of (a) TPPH fluorescence recorded in the absence (black) and presence (red) of GO (0.027 mg ml^{-1}); λ_{ex}= 405 nm, λ_{em}= 653 nm, (b) TPPH fluorescence recorded in the absence (black) and presence (red) of RGO (0.027 mg ml^{-1}); λ_{ex}= 405 nm, λ_{em}= 653 nm.

7. Fluorescence excitation spectra recorded for the porphyrins solution after addition of graphene materials

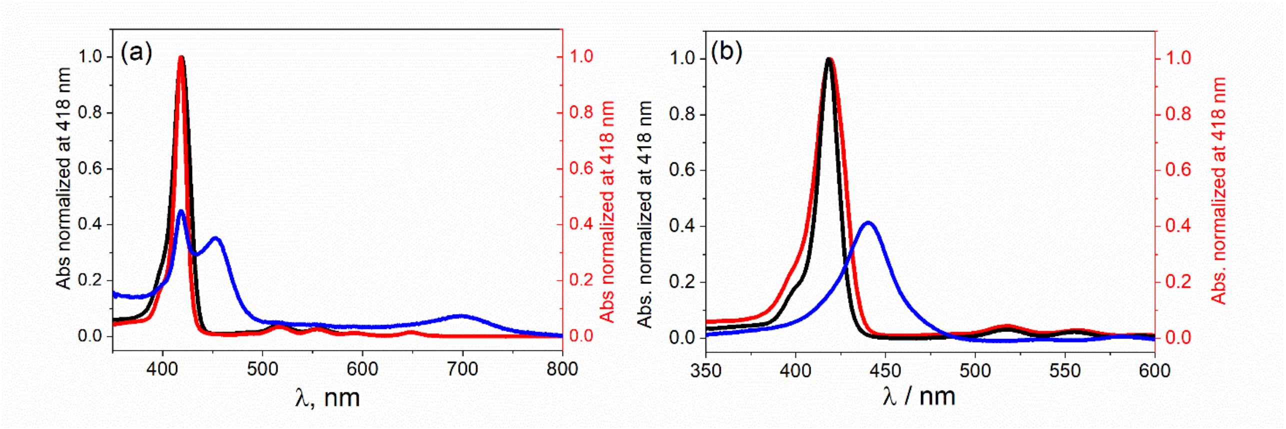

Figure S10. (a) Normalized fluorescence excitation spectrum of the mixture of TPPH (0.8 μM) and GO (0.02 mg ml^{-1}) (red), absorption spectrum of this mixture (blue) and normalized absorption spectrum of TPPH (0.8 μM) in the absence of GO (black); (b) normalized fluorescence excitation spectrum of the mixture of TPPH (0.8 μM) and RGO (0.015 mg ml^{-1}) (red), absorption spectrum of this mixture (blue) and normalized absorption spectrum of TPPH (0.8 μM) in the absence of RGO (black).

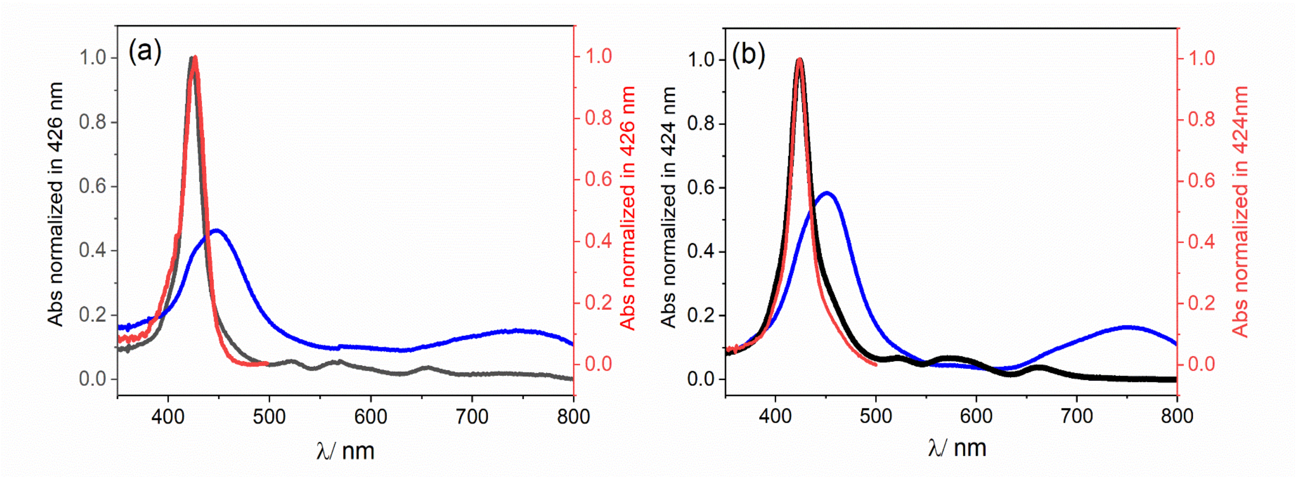

Figure S11. (a) Normalized fluorescence excitation spectrum of the mixture of TAPP (0.8 μM) and GO (0.033 mg ml^{-1}) (red), absorption spectrum of this mixture (blue) and normalized absorption spectrum of TAPP (0.8 μM) in the absence of GO (black); (b) normalized fluorescence excitation spectrum of the mixture of TAPP (0.8 μM) and RGO (0.0 mg ml^{-1}) (red), absorption spectrum of this mixture (blue) and normalized absorption spectrum of TPPH (0.8 μM) in the absence of RGO (black).

8. Covalent vs. non-covalent functionalization

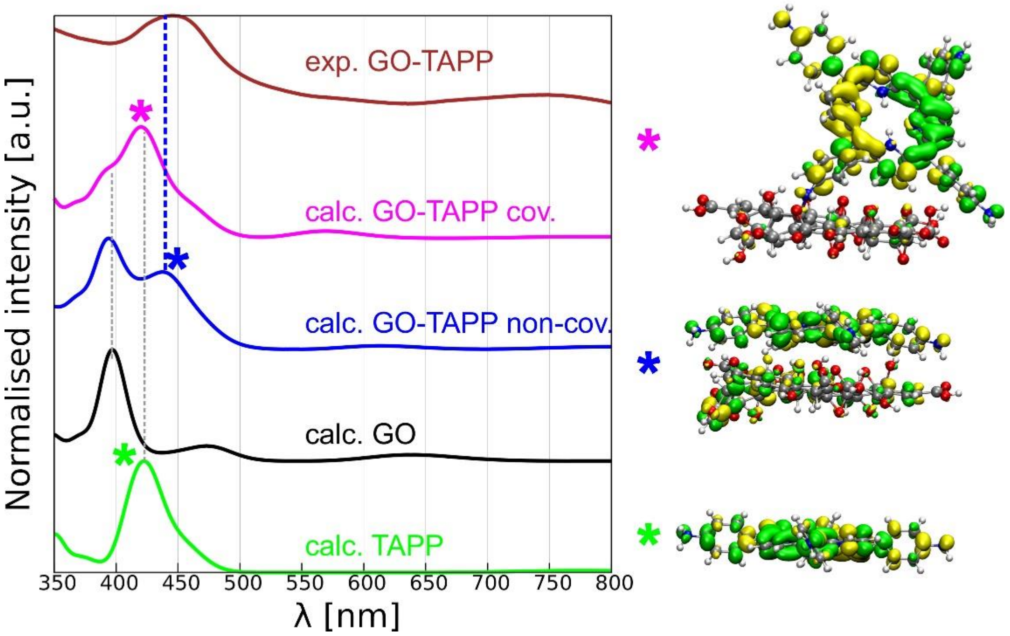

Fig. S12. Left: comparison of experimental GO-TAPP nanohybrid UV-Vis spectrum (brown) with computed UV-Vis spectra for isolated TAPP (green), GO model (black), non-covalently functionalized GO-TAPP nanohybrid (blue) and TAPP linked covalently to the GO model (magenta). Right: transition density isosurfaces (±0.001 a.u.) for Soret bands in three TAPP-containing species. Covalent functionalization is obtained via epoxy ring-opening reaction. In this case, a T-shaped complex is formed and the mixing of the π-orbitals of the TAPP with GO orbitals is limited compared to a system where a non-covalent π-π stacking is present.

9. Canonical MO-based peak decomposition analysis of the absorption spectra.

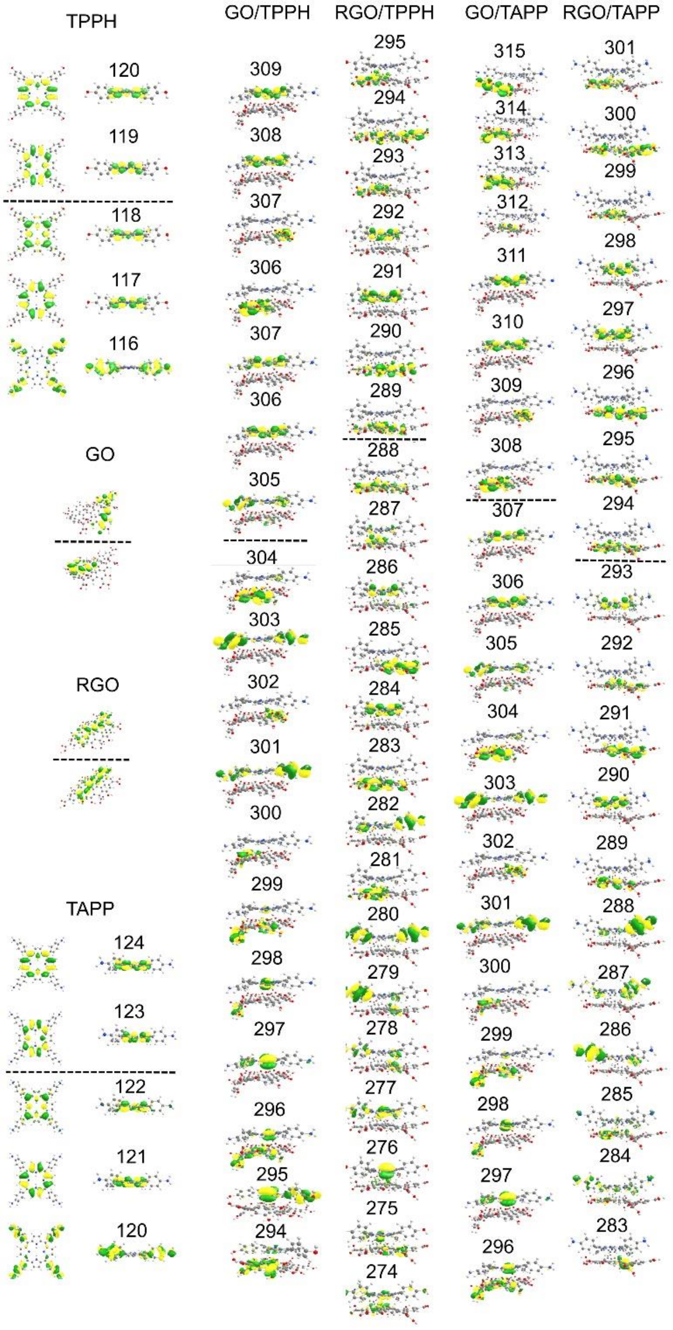

Fig. S13. Isosurfaces (±0.03 a.u.) of key molecular orbitals of the systems studied with the numbers in respect to the Table S1.

Table S1. Unshifted peak decomposition into one-electron excitation contributions (in a form wavelength [nm]: dominant transitions).

	**Soret bands**	**Q bands**
TPPH	340.6 : 117-> 119, 113-> 120<br>305.7 : 117-> 120, 118-> 119<br>288.7 : 117-> 119<br>279.4 : 114-> 119	654 : 117->120, 118->119<br>670 : 113->120, 118->120
GO/TPPH	338.7 : 298-304, 292-304<br>323.0 : 303-> 309, 302-> 308<br>320.4 : 292-> 304<br>303.9 : 302-> 306<br>302.6 : 303-> 312<br>300.3 : 303-> 309, 298-> 304<br>288.5 : 296-> 306<br>285.1 : 295-> 308	689.4 : 303->307, 302->306, 303->308<br>686.0 : 303->306
RGO/TPPH	347.7 : 287-> 295, 283-> 292<br>346.1 : 283-> 290, 284-> 291<br>336.8 : 284-> 293<br>329.7: 0.64 (283-> 291) 0.29 (283-> 293) 0.22 (277-> 292)<br>329.1 : 0.58 (287-> 294) 0.41 (286-> 294)<br>326.8 : 0.30 (283-> 293) 0.28 (288-> 297) 0.28 (276-> 292)<br>324.1 : 0.32 (286-> 294) 0.31 (281-> 293)<br>321.5 : 0.45 (286-> 294) 0.27 (285-> 294)<br>316.4 : 0.34 (288-> 301) 0.31 (274-> 289)<br>313.3 : 0.25 (283-> 292) 0.24 (274-> 289)<br>310.3 : 0.29 (283-> 294)<br>307.0 : 0.34 (283-> 292) 0.28 (276-> 289) 0.24 (277-> 292)<br>301.3 : 0.52 (282-> 289) 0.22 (288-> 300) 0.21 (283-> 293)<br>299.0 : 0.38 (276-> 289) 0.35 (275-> 289)<br>295.8 : 0.33 (285-> 296) 0.25 (284-> 294)<br>292.4 : 0.43 (282-> 291) 0.38 (286-> 298) 0.21 (287-> 298)<br>291.3 : 0.28 (281-> 293)	679.2 : 284-> 291<br>669.9 : 286-> 292
TAPP	340.8 : 0.62 (115-> 124) -0.54 (121-> 123) 0.36 (122-> 124)<br>330.1 : 0.89 (116-> 124) -0.29 (115-> 125) -0.24 (122-> 125)<br>312.7 : 0.65 (121-> 124) 0.55 (122-> 123) -0.23 (117-> 123)<br>298.0 : 0.39 (122-> 124)<br>288.5 : 0.59 (120-> 123) -0.56 (122-> 125) -0.29 (118-> 124)<br>286.0 : 0.61 (120-> 124) -0.53 (118-> 123) 0.42 (116-> 123)	670.0 : -0.76 (122-> 124) -0.61 (121-> 123)<br>655.1 : 0.75 (122-> 123) 0.10 (117-> 123)
GO/TAPP	376.8 : -0.83 (302-> 309) 0.20 (302-> 310) 0.19 (307-> 309)<br>354.1 : -0.56 (306-> 310) 0.44 (307-> 311) -0.27 (307-> 312)<br>344.9 : 0.93 (307-> 312) -0.17 (306-> 310) -0.14 (297-> 311)<br>338.0 : -0.46 (303-> 308) -0.43 (299-> 308) -0.34 (307-> 313)<br>334.0 : -0.37 (299-> 308) -0.36 (298-> 311) 0.35 (303-> 308)<br>331.1 : -0.56 (298-> 311) 0.33 (297-> 311) 0.25 (299-> 311)<br>325.8 : -0.57 (306-> 311) -0.40 (307-> 310) 0.30 (299-> 308)<br>322.7 : 0.95 (306-> 309) -0.21 (306-> 310) 0.10 (302-> 309)<br>312.8 : 0.56 (296-> 308) 0.46 (299-> 308) 0.34 (298-> 308)<br>308.6 : 0.50 (307-> 313) -0.39 (300-> 312) -0.27 (297-> 311)<br>307.2 : -0.53 (307-> 313) -0.46 (300-> 312) 0.25 (303-> 308)<br>305.9 : 0.40 (297-> 311) 0.31 (306-> 312) 0.29 (307-> 313)<br>302.1 : 0.91 (306-> 312) -0.17 (297-> 311) 0.10 (305-> 312)<br>297.6 : 0.47 (305-> 310) -0.43 (307-> 316) 0.34 (300-> 312)<br>292.3 : 0.72 (305-> 309) 0.38 (304-> 309) -0.26 (303-> 309)	684.6 : -0.61 (291-> 295) 0.38 (288-> 296) -0.33 (292-> 297)<br>665.6 : -0.48 (292-> 297) 0.43 (291-> 295) -0.37 (291-> 296)
RGO/TAPP	371.6 -0.53 (288-> 294) -0.36 (290-> 294) -0.31 (287-> 294)<br>368.0 0.44 (289-> 295) 0.42 (289-> 298) -0.27 (291-> 298)<br>359.7 -0.61 (281-> 293) -0.29 (285-> 293) 0.25 (286-> 293)<br>355.3 0.36 (289-> 297) 0.34 (288-> 296) 0.24 (289-> 296)<br>348.8 0.35 (290-> 299) 0.35 (288-> 297) 0.27 (292-> 300)<br>347.2 -0.37 (288-> 297) -0.37 (288-> 296) 0.26 (289-> 297)<br>344.1 -0.38 (288-> 297) -0.35 (288-> 295) 0.32 (281-> 293)<br>339.1 -0.47 (287-> 296) -0.40 (280-> 296) 0.31 (291-> 298)<br>338.2 0.57 (291-> 298) -0.32 (292-> 301) 0.25 (286-> 293)<br>334.4 0.31 (290-> 298) -0.27 (291-> 298) 0.24 (286-> 293)	684.6 -0.61 (291-> 295) 0.38 (288-> 296) -0.33 (292-> 297)<br>665.6 -0.48 (292-> 297) 0.43 (291-> 295) -0.37 (291-> 296)

	334.0 -0.32 (287-> 297) 0.31 (283-> 293) 0.28 (288-> 297)<br>330.4 -0.40 (287-> 297) 0.33 (288-> 297) 0.26 (286-> 293)<br>326.2 0.38 (287-> 295) 0.33 (289-> 297) 0.27 (292-> 301)<br>325.1 -0.69 (287-> 295) 0.26 (292-> 301) 0.17 (291-> 298)<br>322.1 -0.41 (290-> 298) -0.38 (280-> 293) 0.30 (287-> 296)<br>319.5 0.34 (287-> 297) 0.31 (288-> 297) -0.29 (285-> 293)<br>319.1 0.51 (279-> 293) 0.29 (280-> 293) 0.26 (286-> 293)<br>316.9 0.36 (285-> 293) 0.29 (286-> 293) -0.26 (286-> 294)<br>314.4 0.29 (290-> 299) 0.29 (279-> 296) -0.22 (292-> 303)<br>312.5 0.42 (285-> 293) 0.41 (286-> 293) -0.27 (286-> 294)<br>309.2 0.31 (284-> 293) 0.28 (287-> 296) -0.25 (280-> 296)<br>307.5 -0.38 (292-> 303) 0.31 (287-> 296) -0.25 (292-> 304)<br>304.4 -0.48 (284-> 293) -0.31 (287-> 297) 0.28 (286-> 294)<br>302.8 0.47 (284-> 293) 0.34 (286-> 295) 0.33 (291-> 302)<br>300.5 0.37 (292-> 305) -0.21 (287-> 298) 0.20 (287-> 296)	

10. Cartesian coordinates of the optimized structures.

Graphene Oxide

C	20.613480	-16.766376	-1.496904
C	19.163268	-16.768426	-1.201820
C	21.227079	-17.921006	-1.855115
C	18.440522	-18.102649	-1.083019
C	20.528734	-19.181037	-1.936420
C	19.219006	-19.299811	-1.600581
C	20.522774	-14.214526	-1.471577
C	19.240772	-14.461645	-2.255349
C	21.384565	-15.492168	-1.295676
C	18.424462	-15.545827	-1.570827
C	20.499330	-11.628150	-1.970294
C	19.139754	-11.863138	-2.604358
C	21.171162	-12.874542	-1.420731
C	18.467010	-13.194846	-2.596066
C	20.567688	-9.461682	-3.183668
C	19.092995	-9.366012	-2.976249
C	21.228629	-10.628452	-2.833102
C	18.331502	-10.593741	-2.683949
C	20.562015	-7.246990	-4.222045
C	19.118443	-7.225140	-4.179940
C	21.242191	-8.348978	-3.764768
C	18.407220	-8.234185	-3.623843
C	16.917086	-8.252201	-3.706172
C	16.160542	-9.549879	-3.543560
C	16.962432	-10.814044	-3.276241
C	16.266470	-11.906897	-2.498698
C	16.986704	-13.218584	-2.450098
C	16.303773	-14.346816	-2.075908
C	16.962287	-15.605531	-1.809940
C	16.299402	-16.810948	-1.685749
C	16.993295	-18.069165	-1.554450
C	16.367245	-19.280257	-1.746690
C	18.479269	-20.547144	-1.555579
C	17.106314	-20.496467	-1.561718
C	14.818180	-16.858516	-1.675388
C	14.102857	-18.133857	-1.990452
C	14.907653	-19.428068	-2.121778
C	14.828309	-14.292261	-1.898306
C	14.095728	-15.560377	-1.757021
C	14.807822	-11.854885	-2.361616
C	14.054069	-13.141370	-2.521745
C	14.721691	-9.547354	-3.233250
C	14.072793	-10.634213	-2.757718
C	12.803027	-18.070767	-2.692442
C	12.507510	-19.005154	-3.629489
C	14.620371	-20.179690	-3.407173
C	13.428449	-20.038710	-4.031585
C	12.684859	-15.512244	-2.270777
C	12.042549	-16.788457	-2.704747
C	12.595246	-13.087467	-2.094549

C	11.954374	-14.270968	-1.931352
C	12.648075	-10.618434	-2.449041
C	11.955468	-11.765975	-2.038270
C	22.091867	-15.479367	0.068000
O	23.037489	-14.752005	0.344168
O	21.600211	-16.365377	0.945366
C	19.164213	-21.859839	-1.564233
O	20.358595	-22.052315	-1.742879
O	18.309007	-22.907226	-1.345910
O	22.576257	-17.902965	-2.107598
O	22.508585	-12.692763	-1.233403
O	22.487844	-10.904346	-3.191185
O	18.770914	-15.805479	-0.174426
O	20.384153	-13.378204	-0.301517
O	19.028091	-12.605302	-3.832425
C	21.249721	-6.069557	-4.778068
O	20.690959	-5.070115	-5.202293
O	22.617473	-6.189241	-4.782798
C	16.184756	-6.965358	-3.370047
O	15.234017	-6.523381	-3.977926
O	16.716324	-6.385790	-2.264983
C	10.552647	-11.693546	-1.597520
O	9.873191	-12.573797	-1.104694
O	9.966727	-10.434406	-1.781060
C	10.538647	-16.906025	-2.612437
O	9.980804	-17.681534	-1.864277
O	9.886002	-16.042853	-3.431456
O	15.578738	-21.071695	-3.775877
O	14.188824	-17.667346	-0.628519
O	12.593180	-15.871436	-3.689988
O	14.362814	-14.733405	-0.604022
O	12.110042	-9.397455	-2.564201
O	15.648937	-11.616618	-1.198855
O	16.406429	-8.947234	-4.852877
O	18.507669	-9.665609	-1.612324
H	18.362046	-18.280779	0.014196
H	21.085762	-20.080108	-2.202664
H	19.538223	-14.882183	-3.233569
H	22.205440	-15.442078	-2.031110
H	20.250678	-11.057561	-1.056957
H	18.620515	-6.364852	-4.629991
H	22.334354	-8.326264	-3.805168
H	17.147358	-11.257636	-4.268950
H	16.552940	-21.431544	-1.520709
H	14.463996	-20.078456	-1.334897
H	14.059780	-13.329947	-3.614860
H	14.171286	-8.641467	-3.482603
H	11.581609	-18.907497	-4.199408
H	13.179615	-20.667160	-4.889932
H	10.905813	-14.315256	-1.661535
H	22.104727	-16.238171	1.776968
H	18.874998	-23.705293	-1.380839
H	22.858601	-18.801449	-2.353405
H	22.860937	-13.429049	-0.655511

H	22.795164	-10.213702	-3.812884
H	22.942638	-5.351388	-5.170172
H	16.179520	-5.583280	-2.094057
H	9.064573	-10.507072	-1.408113
H	8.932806	-16.167973	-3.235926
H	15.273555	-21.545939	-4.572113
H	11.148988	-9.453576	-2.326328

Reduced Graphene Oxide (deepoxy)

C	20.659872	-16.718814	-1.819413
C	19.222992	-16.730232	-1.911040
C	21.336323	-17.905321	-1.879399
C	18.525537	-18.014442	-1.565583
C	20.669658	-19.180598	-1.997792
C	19.325066	-19.278236	-1.826330
C	20.620675	-14.183597	-1.890197
C	19.290985	-14.352159	-2.615732
C	21.360839	-15.442834	-1.456005
C	18.519936	-15.592359	-2.198682
C	20.501438	-11.689998	-2.062369
C	19.088180	-11.849201	-2.584916
C	21.190141	-12.970255	-1.706227
C	18.459884	-13.089936	-2.643724
C	20.595992	-9.487936	-3.190896
C	19.139867	-9.458628	-3.059911
C	21.258774	-10.670509	-2.867870
C	18.430860	-10.643396	-2.882345
C	20.566411	-7.189261	-3.989404
C	19.153776	-7.132865	-3.795330
C	21.267357	-8.340108	-3.690213
C	18.436649	-8.222443	-3.333513
C	16.990539	-8.197836	-3.140401
C	16.266392	-9.387158	-3.077617
C	16.999752	-10.692977	-3.318354
C	16.279522	-11.952074	-2.897278
C	17.040109	-13.166800	-2.740379
C	16.344275	-14.401160	-2.543985
C	17.072337	-15.612603	-2.269705
C	16.372130	-16.827649	-2.138119
C	17.070723	-18.068208	-1.944827
C	16.436379	-19.292697	-1.953835
C	18.574958	-20.521934	-1.693263
C	17.198601	-20.476608	-1.707692
C	14.937378	-16.867652	-2.257808
C	14.237277	-18.065619	-2.286069
C	14.932816	-19.391608	-2.066173
C	14.918713	-14.437403	-2.572039
C	14.221913	-15.627942	-2.388641
C	14.912011	-11.931947	-2.826500
C	14.128773	-13.202745	-2.942404
C	14.868887	-9.463708	-2.872929
C	14.203905	-10.682268	-2.801543

C	12.819127	-18.087570	-2.497777
C	12.231838	-19.285805	-2.915522
C	14.354565	-20.449659	-2.985962
C	13.020789	-20.411751	-3.273475
C	12.777277	-15.628202	-2.336680
C	12.092443	-16.839259	-2.411730
C	12.729614	-13.166550	-2.354392
C	12.117689	-14.362093	-2.123740
C	12.771324	-10.693216	-2.562588
C	12.065495	-11.870878	-2.257658
C	21.552893	-15.376604	0.063669
O	22.461350	-14.752982	0.604938
O	20.622672	-16.033458	0.787327
C	19.258475	-21.819463	-1.570340
O	20.465404	-22.018349	-1.656606
O	18.398155	-22.866454	-1.337476
O	22.697380	-17.896557	-1.751892
O	22.381025	-12.730785	-1.097817
O	22.533747	-10.943795	-3.183575
C	21.239942	-5.972912	-4.491512
O	20.680488	-4.925931	-4.771417
O	22.597903	-6.124321	-4.628358
C	16.279323	-6.898964	-3.015243
O	15.167702	-6.636201	-3.446860
O	16.999383	-5.971748	-2.307959
C	10.662050	-11.821740	-1.819144
O	10.003812	-12.692382	-1.279057
O	10.035950	-10.590117	-2.066546
C	10.607153	-16.908184	-2.409702
O	9.942978	-17.749680	-1.825630
O	10.015640	-15.943469	-3.178399
O	15.195476	-21.439601	-3.382910
O	12.220810	-9.470965	-2.588124
H	18.478739	-17.990794	-0.442220
H	21.262492	-20.090758	-2.093057
H	19.551445	-14.544327	-3.683186
H	22.382809	-15.431129	-1.868195
H	20.365960	-11.160657	-1.086209
H	18.660996	-6.191164	-4.024323
H	22.354168	-8.324858	-3.798401
H	17.023800	-10.789631	-4.435372
H	16.663520	-21.412798	-1.571449
H	14.572869	-19.732474	-1.060461
H	13.943480	-13.299704	-4.042651
H	14.300325	-8.545710	-2.765539
H	11.158494	-19.325119	-3.087565
H	12.555841	-21.237206	-3.819667
H	11.090450	-14.370641	-1.782339
H	20.835444	-15.853330	1.727779
H	18.979494	-23.651802	-1.283958
H	23.016131	-18.816201	-1.795823
H	22.649261	-13.497745	-0.522526
H	22.887318	-10.217708	-3.735390
H	22.915314	-5.261195	-4.963441

H	16.438726	-5.167426	-2.303239
H	9.146784	-10.667773	-1.664297
H	9.051579	-16.074037	-3.054223
H	14.689851	-22.082761	-3.915258
H	11.252725	-9.558351	-2.394031

Reduced Graphene Oxide (graphene)

C	20.641997	-16.730191	-1.920235
C	19.205797	-16.758878	-2.016634
C	21.339556	-17.972396	-1.746578
C	18.510209	-17.995589	-1.938259
C	20.664977	-19.157951	-1.668854
C	19.237054	-19.212590	-1.759440
C	20.611999	-14.286440	-2.167883
C	19.173054	-14.311726	-2.263897
C	21.308620	-15.507718	-1.998336
C	18.482302	-15.544681	-2.187870
C	20.577997	-11.836173	-2.412545
C	19.139327	-11.862050	-2.509950
C	21.276614	-13.050769	-2.244033
C	18.450624	-13.094401	-2.433944
C	20.544792	-9.385551	-2.658644
C	19.111831	-9.411272	-2.758912
C	21.242969	-10.597429	-2.487867
C	18.417537	-10.646529	-2.682983
C	20.497126	-6.956439	-2.906522
C	19.105837	-6.974874	-3.006193
C	21.214548	-8.137271	-2.734115
C	18.381585	-8.191128	-2.934979
C	16.971567	-8.236141	-3.031961
C	16.264340	-9.441404	-2.959346
C	16.994211	-10.671180	-2.781057
C	16.299742	-11.904686	-2.704843
C	17.023628	-13.120810	-2.529437
C	16.330156	-14.355643	-2.453886
C	17.051787	-15.573034	-2.278246
C	16.358905	-16.803210	-2.195591
C	17.083169	-18.024612	-2.026218
C	16.395523	-19.262678	-1.937859
C	18.519392	-20.422242	-1.652184
C	17.136872	-20.443368	-1.720490
C	14.930632	-16.827690	-2.291288
C	14.233135	-18.065696	-2.218737
C	14.947238	-19.300148	-2.056058
C	14.906187	-14.380126	-2.557166
C	14.213856	-15.615886	-2.486333
C	14.873795	-11.933594	-2.804267
C	14.185070	-13.159969	-2.731675
C	14.844019	-9.500254	-3.056834
C	14.150509	-10.696942	-2.985091
C	12.804916	-18.087620	-2.377317
C	12.131911	-19.334619	-2.374585

C	14.221945	-20.487185	-2.055042
C	12.840797	-20.523240	-2.203534
C	12.776246	-15.640327	-2.602311
C	12.112897	-16.868192	-2.549572
C	12.743905	-13.194281	-2.840566
C	12.083055	-14.408784	-2.777890
C	12.713977	-10.761115	-3.086172
C	12.047664	-11.945664	-3.017915
H	21.209323	-20.094340	-1.531745
H	18.554890	-6.042517	-3.142288
H	22.303138	-8.116251	-2.655433
H	16.573968	-21.371387	-1.600731
H	14.291987	-8.567738	-3.194547
H	11.043687	-19.347638	-2.486501
H	12.292607	-21.469657	-2.164414
H	10.993659	-14.431957	-2.866090
H	19.073073	-21.350134	-1.493951
H	22.428942	-17.947956	-1.675675
H	22.366775	-13.027597	-2.170516
H	22.333066	-10.574582	-2.411842
H	21.028601	-6.005554	-2.963687
H	12.167693	-9.825873	-3.222655
H	10.958771	-11.973288	-3.098843
H	22.398939	-15.486613	-1.926997
H	16.422372	-7.302159	-3.169139
H	11.025276	-16.890881	-2.656878

Reduced Graphene Oxide (defective)

C	20.643921	-16.741602	-2.382535
C	19.210071	-16.656904	-2.229894
C	21.315820	-17.878244	-2.008197
C	18.511176	-17.754636	-1.458648
C	20.630701	-19.050830	-1.553147
C	19.274504	-19.058272	-1.367111
C	20.620528	-14.310045	-2.867770
C	19.179565	-14.384363	-3.295177
C	21.359555	-15.599420	-3.069452
C	18.482153	-15.588255	-2.680967
C	20.375141	-11.963438	-2.030708
C	19.038857	-11.910224	-2.745284
C	21.155913	-13.218950	-2.314097
C	18.402444	-13.107580	-3.105729
C	20.510440	-9.488840	-2.321450
C	19.077430	-9.458012	-2.645851
C	21.146266	-10.689413	-2.125244
C	18.401369	-10.669848	-2.887516
C	20.559670	-7.048701	-2.394725
C	19.168849	-7.027732	-2.709174
C	21.215314	-8.236313	-2.227047
C	18.444857	-8.193563	-2.853648
C	17.001005	-8.126789	-3.305751
C	16.242105	-9.416985	-3.230621

C	17.027446	-10.665852	-3.529584
C	16.256180	-11.943762	-3.293043
C	16.998485	-13.166588	-3.225063
C	16.315556	-14.415914	-3.032065
C	17.028633	-15.581288	-2.647735
C	16.319812	-16.750524	-2.272032
C	17.035751	-17.901499	-1.770634
C	16.399049	-19.068414	-1.424568
C	18.529872	-20.209941	-0.966005
C	17.162579	-20.196471	-0.948041
C	14.898385	-16.813756	-2.419095
C	14.195106	-17.999170	-2.131457
C	14.889230	-19.150425	-1.433807
C	14.887011	-14.453350	-3.076411
C	14.188215	-15.625680	-2.828168
C	14.883545	-11.930548	-3.247230
C	14.120800	-13.213198	-3.463405
C	14.908619	-9.473149	-3.016787
C	14.154912	-10.706442	-3.087487
C	12.817577	-18.139980	-2.423456
C	12.217576	-19.387488	-2.376468
C	14.294979	-20.490382	-1.784574
C	12.996058	-20.570967	-2.162936
C	12.737600	-15.636602	-2.863361
C	12.022085	-16.955108	-2.911947
C	12.690144	-13.192639	-2.970549
C	12.042985	-14.449779	-2.842436
C	12.775055	-10.749248	-2.903132
C	12.066933	-11.972555	-2.803813
H	18.517562	-17.386673	-0.397858
H	21.200802	-19.964206	-1.369607
H	19.181833	-14.567159	-4.395109
H	21.406472	-15.820606	-4.154412
H	20.092765	-12.030185	-0.946315
H	18.671669	-6.067208	-2.861155
H	22.280520	-8.261473	-1.990446
H	17.239782	-10.650881	-4.629431
H	16.631949	-21.091031	-0.623571
H	14.608605	-19.012871	-0.351922
H	14.002432	-13.274492	-4.577948
H	14.357826	-8.552392	-2.802817
H	11.161042	-19.482138	-2.635059
H	12.539744	-21.544769	-2.350051
H	10.956874	-14.465840	-2.709070
H	14.897279	-21.388291	-1.657790
H	19.074448	-21.122403	-0.713650
H	22.398774	-17.922463	-2.145858
H	22.202215	-13.227646	-1.993796
H	22.219566	-10.714670	-1.923119
H	21.097672	-6.104834	-2.294905
H	12.238598	-9.811370	-2.746453
H	11.001492	-11.952971	-2.563191
H	22.396977	-15.530062	-2.712240
H	17.010284	-7.799852	-4.365080

H	16.464263	-7.337085	-2.758288
H	11.727842	-17.150704	-3.961621
H	11.071908	-16.896267	-2.355766

TPPH

C	-0.142176	-1.325342	-2.604983
C	-0.163382	2.142704	0.843261
C	-0.209568	-1.330049	4.336382
C	-0.161268	-4.798248	0.888378
N	-0.159234	0.173176	2.358506
N	-0.174655	0.106011	-0.576407
N	-0.128908	-2.828567	-0.626458
N	-0.202013	-2.761354	2.307481
C	-0.220710	-4.180093	-0.372525
C	-0.213200	-2.582173	-1.979917
C	-0.066783	-0.086727	-1.931403
C	-0.075079	1.462759	-0.390877
C	-0.098665	-4.118177	2.124033
C	-0.121364	-2.568742	3.664430
C	-0.270448	-0.073406	3.709982
C	-0.248041	1.524541	2.102610
C	-0.392393	-4.824835	-1.642617
C	-0.388386	-3.855143	-2.618029
C	0.147241	1.181407	-2.621144
C	0.142544	2.146415	-1.661632
C	-0.447020	2.169024	3.368847
C	-0.461199	1.199284	4.344195
C	0.092921	-4.801796	3.399022
C	0.078173	-3.836904	4.358428
H	-0.527028	-5.892787	-1.775163
H	-0.517879	-3.993664	-3.685902
H	0.312837	1.303988	-3.687046
H	0.302061	3.212633	-1.789804
H	-0.587218	3.236675	3.498172
H	-0.615108	1.336970	5.408869
H	0.248725	-5.868114	3.530736
H	0.219507	-3.960195	5.427796
C	-0.154221	-1.316748	-4.091889
C	0.855807	-1.954525	-4.833114
C	-1.179632	-0.668882	-4.797820
H	1.668364	-2.450855	-4.301068
H	-1.977005	-0.175757	-4.241031
C	0.850458	-1.946805	-6.225177
C	-1.200843	-0.658108	-6.191921
H	1.643514	-2.430534	-6.795649
H	-2.016126	-0.158346	-6.722114
C	-0.183433	-1.297570	-6.911155
C	-0.174090	3.629787	0.827131
C	-1.194351	4.337749	0.167982
C	0.832812	4.368512	1.466912
H	-1.989273	3.778252	-0.326207
H	1.640962	3.838718	1.972592

C	-1.215262	5.729374	0.146245
C	0.827862	5.762960	1.448075
H	-2.014449	6.274295	-0.356532
H	1.630780	6.319453	1.939073
C	-0.199056	6.448599	0.787186
C	-0.245048	-1.338409	5.822949
C	-1.281743	-1.987355	6.516668
C	0.753408	-0.702219	6.576005
H	-2.070318	-2.479190	5.946206
H	1.574497	-0.204933	6.058275
C	-1.326668	-2.002236	7.907679
C	0.724263	-0.714099	7.970256
H	-2.138425	-2.497262	8.440991
H	1.521012	-0.225690	8.537763
C	-0.318721	-1.364474	8.641422
C	-0.171052	-6.285220	0.904059
C	0.846762	-7.022913	0.280457
C	-1.201527	-6.994258	1.546022
H	1.662951	-6.492538	-0.211504
H	-2.004853	-6.435830	2.027650
C	0.842302	-8.417308	0.298209
C	-1.221919	-8.385867	1.566415
H	1.653460	-8.972835	-0.180138
H	-2.028850	-8.931481	2.055865
C	-0.194992	-9.104054	0.941549
H	-0.090027	-0.550333	1.640400
H	-0.077969	-2.105091	0.093181
O	-0.147330	-1.319784	-8.282265
H	-0.921642	-0.836583	-8.621114
O	-0.256435	-10.473583	0.991501
H	0.516250	-10.836106	0.523163
O	-0.404485	-1.409092	10.009783
H	0.358939	-0.935131	10.384381
O	-0.260380	7.818146	0.734973
H	0.504928	8.181473	1.214662

TAPP

C	-0.127735	-1.328720	-2.602474
C	-0.153631	2.144437	0.844793
C	-0.194303	-1.327767	4.341216
C	-0.172403	-4.800884	0.893885
N	-0.148366	0.175549	2.361971
N	-0.164527	0.105043	-0.573911
N	-0.128421	-2.832070	-0.622776
N	-0.199302	-2.761345	2.312210
C	-0.242884	-4.181781	-0.366839
C	-0.222587	-2.584035	-1.975536
C	-0.034807	-0.091381	-1.926669
C	-0.046368	1.460170	-0.386820
C	-0.086196	-4.117251	2.127438
C	-0.095555	-2.565727	3.667349
C	-0.270224	-0.072058	3.712572

C	-0.252256	1.525697	2.103868
C	-0.444531	-4.822569	-1.634086
C	-0.432280	-3.853129	-2.610214
C	0.218759	1.171364	-2.611760
C	0.211837	2.137590	-1.652806
C	-0.476393	2.167323	3.366926
C	-0.487139	1.197875	4.343072
C	0.142031	-4.796262	3.398311
C	0.136322	-3.830034	4.357252
H	-0.604079	-5.887497	-1.763249
H	-0.580003	-3.987698	-3.676195
H	0.410805	1.288016	-3.673860
H	0.396981	3.200174	-1.776164
H	-0.634460	3.232854	3.492904
H	-0.655479	1.333002	5.405886
H	0.317427	-5.860102	3.525301
H	0.306132	-3.947970	5.423042
C	-0.131121	-1.319638	-4.087508
C	0.847339	-2.006054	-4.826401
C	-1.114237	-0.617436	-4.805626
H	1.631576	-2.544743	-4.292738
H	-1.889884	-0.084658	-4.254276
C	0.851708	-1.990331	-6.217979
C	-1.124123	-0.600455	-6.196715
H	1.633897	-2.521876	-6.764851
H	-1.911423	-0.060787	-6.728233
C	-0.136956	-1.285535	-6.929290
C	-0.168523	3.629244	0.824757
C	-1.151346	4.334492	0.109242
C	0.798569	4.380906	1.513538
H	-1.918656	3.773262	-0.425303
H	1.582352	3.857544	2.062916
C	-1.171603	5.725337	0.081248
C	0.792439	5.772280	1.487043
H	-1.958498	6.246490	-0.469049
H	1.565907	6.329295	2.020780
C	-0.195589	6.470647	0.768479
C	-0.225462	-1.336399	5.825923
C	-1.226150	-2.031897	6.525956
C	0.743880	-0.656390	6.582593
H	-1.995369	-2.559285	5.960581
H	1.541148	-0.122611	6.063491
C	-1.261469	-2.049011	7.916686
C	0.722851	-0.672383	7.973957
H	-2.061996	-2.583563	8.433435
H	1.498338	-0.145989	8.535150
C	-0.283127	-1.370791	8.667156
C	-0.195077	-6.285627	0.913501
C	0.783342	-7.041903	0.246243
C	-1.196699	-6.986034	1.607333
H	1.581400	-6.522243	-0.285829
H	-1.972900	-6.421177	2.124926
C	0.770260	-8.433210	0.273125
C	-1.224323	-8.376732	1.634926

H	1.552894	-8.994151	-0.242839
H	-2.026171	-8.893678	2.167281
C	-0.236669	-9.126820	0.969717
H	-0.070379	-0.547527	1.644439
H	-0.062975	-2.109242	0.096260
N	-0.243995	7.864599	0.787348
H	-0.730076	8.294004	0.005650
H	0.634334	8.328943	0.997754
N	-0.174975	-1.315683	-8.323316
H	0.708087	-1.523563	-8.779733
H	-0.663814	-0.541199	-8.762538
N	-0.290646	-10.520339	0.950218
H	-0.794930	-10.948435	1.720930
H	0.589081	-10.988862	0.755854
N	-0.345764	-1.340429	10.060323
H	0.530785	-1.139636	10.532203
H	-0.848108	-2.111300	10.490579

GO TPPH

C	12.885363	-18.557591	1.075803
C	11.969014	-17.555890	0.498715
C	12.761438	-19.858588	0.711221
C	10.887777	-18.025812	-0.480208
C	11.727932	-20.347054	-0.165683
C	10.854220	-19.508398	-0.768617
C	13.989935	-16.642605	2.347111
C	13.801415	-15.836855	1.055066
C	13.966690	-18.164849	2.058120
C	12.443213	-16.156546	0.438475
C	14.938351	-14.552620	3.663139
C	14.749346	-13.755153	2.382327
C	14.670574	-16.048663	3.543299
C	14.071996	-14.338551	1.184582
C	16.224131	-12.655803	4.618822
C	15.369245	-11.739539	3.818637
C	16.159862	-14.020419	4.378925
C	14.659883	-12.270115	2.640377
C	17.129136	-10.745982	5.837471
C	16.474310	-9.846457	4.917552
C	17.061180	-12.103880	5.628483
C	15.702077	-10.307198	3.905919
C	15.204836	-9.395466	2.843127
C	14.624877	-9.947704	1.563910
C	14.527905	-11.457351	1.380200
C	13.333771	-11.954618	0.599073
C	13.272248	-13.425048	0.326433
C	12.326031	-13.881100	-0.557050
C	11.952035	-15.271383	-0.653252
C	11.131596	-15.779453	-1.647120
C	10.764310	-17.169624	-1.723705
C	10.139429	-17.709925	-2.827863
C	9.823831	-19.893187	-1.723532

C	9.527523	-18.999921	-2.723618
C	10.572876	-14.888863	-2.690444
C	10.113888	-15.454026	-3.996464
C	9.945187	-16.963214	-4.128934
C	11.624394	-12.909566	-1.439859
C	10.852956	-13.438223	-2.569992
C	12.644527	-11.006797	-0.289246
C	12.219342	-11.525099	-1.628738
C	13.796029	-9.079690	0.713973
C	12.932727	-9.562192	-0.204190
C	10.361167	-14.677066	-5.227394
C	10.733305	-15.335720	-6.353394
C	10.647786	-17.542526	-5.336024
C	10.921369	-16.766138	-6.411336
C	10.851866	-12.568058	-3.792114
C	10.601561	-13.209399	-5.116439
C	11.342004	-10.587375	-2.450641
C	10.691997	-11.126958	-3.512442
C	12.095874	-8.690013	-1.017056
C	11.316796	-9.166378	-2.080534
C	13.852109	-18.995537	3.346862
O	14.810588	-19.240973	4.073334
O	12.620748	-19.411081	3.634833
C	9.072925	-21.160871	-1.707551
O	8.096866	-21.422072	-2.408631
O	9.523598	-22.074647	-0.800125
O	13.677737	-20.757756	1.201705
O	15.396667	-16.736060	4.465208
O	17.093938	-14.868841	4.817216
O	11.440457	-16.537669	1.410946
O	13.224862	-16.233725	3.496618
O	15.530664	-14.078190	1.224137
C	17.736658	-10.184644	7.046876
O	17.469234	-9.078279	7.514153
O	18.622024	-11.018025	7.665248
C	14.775479	-8.004518	3.277979
O	15.089405	-6.969623	2.732587
O	13.968681	-8.089174	4.361859
C	10.449283	-8.246235	-2.837689
O	9.805661	-8.482974	-3.848049
O	10.360019	-6.972082	-2.280877
C	9.996439	-12.351348	-6.204077
O	8.845226	-12.465784	-6.570753
O	10.862989	-11.433398	-6.698963
O	10.824927	-18.888805	-5.278949
O	9.149900	-14.984908	-3.034226
O	11.958974	-12.908226	-4.696753
O	10.181128	-12.927902	-1.393618
O	12.147429	-7.414755	-0.620601
O	11.990587	-11.473985	0.918212
O	15.993212	-9.440844	1.644126
O	13.872728	-11.931709	3.784084
H	9.942305	-17.827798	0.077536
H	11.665838	-21.421577	-0.342816

H	14.566239	-16.195730	0.341845
H	14.958176	-18.432398	1.651291
H	14.099879	-14.200928	4.291354
H	16.646381	-8.776990	5.047669
H	17.564079	-12.761543	6.341823
H	15.398184	-11.735142	0.760857
H	8.808495	-19.297415	-3.486050
H	8.867502	-17.077327	-4.380585
H	13.158473	-11.660812	-2.200904
H	13.957338	-8.006939	0.811852
H	11.005414	-14.757548	-7.238910
H	11.324281	-17.209928	-7.324364
H	10.108385	-10.507337	-4.183766
H	12.656469	-19.758994	4.558994
H	8.912631	-22.836115	-0.885748
H	13.420926	-21.654384	0.925570
H	15.349942	-17.718192	4.310487
H	17.843083	-14.343946	5.166835
H	18.892842	-10.553421	8.483849
H	13.721865	-7.175553	4.617341
H	9.468131	-6.618329	-2.521853
H	10.347121	-10.901308	-7.341582
H	11.183354	-19.197081	-6.132376
H	11.467933	-6.906627	-1.145683
C	10.453642	-16.684112	5.230727
C	7.709121	-16.131889	1.226306
C	8.486529	-11.275857	1.473576
C	11.285056	-11.830595	5.437476
N	8.219514	-13.744092	1.704761
N	9.073351	-16.011773	3.281055
N	10.643858	-14.205130	5.082123
N	9.783615	-11.947145	3.468886
C	11.162326	-13.175319	5.840544
C	10.784936	-15.411783	5.732026
C	9.740993	-16.938385	4.036657
C	8.525289	-16.703677	2.230706
C	10.713612	-11.309159	4.249767
C	9.465726	-11.060741	2.469252
C	7.826384	-12.505574	1.247612
C	7.468450	-14.741371	1.122814
C	11.607035	-13.762786	7.070049
C	11.384623	-15.119787	7.001544
C	9.646525	-18.259322	3.427666
C	8.902795	-18.113071	2.297592
C	6.471167	-14.087681	0.331144
C	6.685504	-12.730275	0.410337
C	11.047734	-10.003648	3.692252
C	10.273217	-9.849165	2.584230
H	12.020289	-13.204523	7.902133
H	11.590882	-15.859270	7.767367
H	10.127575	-19.163720	3.778070
H	8.665446	-18.887189	1.575399
H	5.682276	-14.599984	-0.207850
H	6.100078	-11.945592	-0.053927

H	11.822822	-9.339583	4.058536
H	10.299728	-9.027839	1.875393
C	10.945651	-17.855425	5.996347
C	12.310072	-17.998232	6.309794
C	10.076981	-18.894022	6.372687
H	13.009714	-17.223324	5.998508
H	9.013572	-18.798417	6.151568
C	12.797039	-19.141832	6.942949
C	10.547342	-20.039659	7.014098
H	13.859706	-19.253255	7.158609
H	9.849711	-20.830440	7.302614
C	11.912882	-20.170195	7.299638
C	7.168418	-16.982494	0.142732
C	6.644057	-18.276110	0.350840
C	7.236753	-16.527586	-1.188873
H	6.495091	-18.632876	1.369439
H	7.655296	-15.546594	-1.396309
C	6.306559	-19.102930	-0.717724
C	6.861541	-17.328656	-2.259076
H	5.921323	-20.107734	-0.537407
H	6.950850	-16.962675	-3.282697
C	6.448002	-18.645968	-2.037488
C	8.164351	-10.186643	0.521579
C	7.937774	-8.855120	0.922180
C	8.101145	-10.470573	-0.855720
H	7.936345	-8.613212	1.984955
H	8.318422	-11.477884	-1.202796
C	7.711202	-7.841981	-0.009180
C	7.834859	-9.477594	-1.789630
H	7.554114	-6.811727	0.310400
H	7.823425	-9.720980	-2.852620
C	7.673187	-8.156115	-1.369844
C	12.127839	-10.936218	6.261422
C	13.408714	-11.335052	6.684319
C	11.718218	-9.630500	6.599100
H	13.757948	-12.339474	6.450127
H	10.709485	-9.310790	6.336949
C	14.264684	-10.458150	7.338926
C	12.576082	-8.736081	7.233313
H	15.251152	-10.796798	7.649767
H	12.255351	-7.719474	7.464085
C	13.883663	-9.126972	7.557793
H	8.962937	-13.914252	2.382395
H	10.168551	-14.066436	4.189625
O	12.439585	-21.272463	7.923714
H	11.716584	-21.894223	8.120766
O	14.736116	-8.193736	8.070564
H	15.663472	-8.516509	7.984441
O	7.530930	-7.119678	-2.285636
H	7.488916	-7.522315	-3.175277
O	6.209879	-19.445711	-3.124597
H	6.570417	-20.336826	-2.915943

RGO TPPH

C	9.244631	-11.287324	1.263746
C	12.165239	-11.918495	5.155608
C	10.847680	-16.654616	5.449978
C	7.892806	-16.012175	1.602292
N	11.304211	-14.247766	5.044952
N	10.581421	-12.023955	3.234128
N	8.767360	-13.680438	1.691079
N	9.494329	-15.917676	3.501327
C	7.876497	-14.645654	1.263388
C	8.489879	-12.465296	1.098112
C	10.289544	-11.147730	2.214080
C	11.561537	-11.411751	3.980130
C	8.723368	-16.597151	2.587305
C	10.055724	-16.887074	4.300112
C	11.351955	-15.390039	5.817016
C	11.970441	-13.214241	5.666676
C	6.963019	-13.988214	0.377790
C	7.330488	-12.665959	0.282895
C	11.168543	-9.987207	2.272440
C	11.945475	-10.141389	3.378916
C	12.440928	-13.725347	6.919550
C	12.058864	-15.043942	7.014661
C	8.866563	-18.037503	2.764559
C	9.699890	-18.217465	3.827076
H	6.122151	-14.470897	-0.105076
H	6.829357	-11.888131	-0.280291
H	11.203370	-9.173064	1.559504
H	12.736740	-9.484149	3.717927
H	12.973196	-13.139904	7.660116
H	12.225692	-15.718116	7.846204
H	8.427422	-18.803138	2.134303
H	10.082756	-19.153894	4.218249
C	8.948631	-10.128861	0.395982
C	8.722500	-10.270548	-0.986580
C	8.953177	-8.817666	0.914692
H	8.689209	-11.264753	-1.427003
H	9.016580	-8.673073	1.992526
C	8.626852	-9.165459	-1.824671
C	8.906888	-7.707382	0.082126
H	8.504565	-9.293847	-2.897801
H	8.965239	-6.701649	0.501411
C	8.810289	-7.879685	-1.305059
C	13.151242	-11.064232	5.864498
C	12.862731	-9.739864	6.234214
C	14.446268	-11.550019	6.128258
H	11.861016	-9.345695	6.063120
H	14.692047	-12.572192	5.844448
C	13.833487	-8.927775	6.816994
C	15.428700	-10.749294	6.700239
H	13.586267	-7.902890	7.104502
H	16.435551	-11.133585	6.860770
C	15.123164	-9.426943	7.044481

C	11.163747	-17.792063	6.344165
C	10.158242	-18.665688	6.804095
C	12.478122	-18.028218	6.779518
H	9.125419	-18.489477	6.502570
H	13.280720	-17.382714	6.428240
C	10.451335	-19.732343	7.647869
C	12.786321	-19.085795	7.627136
H	9.666759	-20.400595	8.002620
H	13.817136	-19.255741	7.945172
C	11.772781	-19.948858	8.060879
C	6.981151	-16.903381	0.845061
C	6.972498	-16.903912	-0.559223
C	6.111987	-17.790225	1.510120
H	7.644564	-16.240280	-1.101338
H	6.088608	-17.789266	2.600389
C	6.142731	-17.765665	-1.274254
C	5.275538	-18.651022	0.807699
H	6.185220	-17.776250	-2.367132
H	4.597267	-19.327359	1.328167
C	5.292245	-18.644584	-0.593243
H	10.771551	-14.162833	4.176964
H	9.456335	-13.794700	2.438193
C	15.175309	-9.686660	2.456019
C	14.373704	-10.356207	1.499051
C	15.017061	-8.310928	2.575807
C	13.784628	-9.546905	0.399470
C	14.086066	-7.549613	1.810567
C	13.430856	-8.107029	0.730107
C	15.970828	-11.931552	3.324839
C	14.614611	-12.399726	2.841571
C	16.264533	-10.438473	3.171523
C	14.151374	-11.725793	1.574066
C	16.500792	-14.197062	4.260998
C	15.188445	-14.689684	3.675839
C	16.800483	-12.744515	4.017515
C	14.412780	-13.884929	2.846882
C	16.366178	-16.064788	5.891259
C	15.553741	-16.767064	4.897512
C	16.665874	-14.722874	5.665186
C	14.883134	-16.038687	3.917605
C	16.420141	-18.084216	7.248493
C	15.732539	-18.801562	6.224637
C	16.736726	-16.750735	7.080259
C	15.280141	-18.176165	5.078151
C	14.437641	-18.852182	4.096268
C	13.642593	-18.116598	3.220746
C	13.632129	-16.610481	3.334310
C	13.091277	-15.852855	2.151462
C	13.407618	-14.458009	2.015278
C	12.816805	-13.716102	0.942555
C	13.254830	-12.367195	0.687485
C	12.697816	-11.654400	-0.422395
C	12.887119	-10.267841	-0.537677
C	12.259064	-9.504119	-1.548582

C	12.541043	-7.447679	-0.190406
C	12.063031	-8.149981	-1.328315
C	11.753283	-12.295096	-1.308068
C	11.269517	-11.631968	-2.431161
C	11.898188	-10.294017	-2.795375
C	11.835795	-14.309436	0.100330
C	11.317892	-13.627486	-1.006518
C	12.192232	-16.476719	1.325804
C	11.272330	-15.662391	0.468229
C	12.745691	-18.682647	2.282666
C	12.001748	-17.895836	1.412896
C	10.269805	-12.220156	-3.267133
C	9.758730	-11.474324	-4.335674
C	11.200537	-9.572636	-3.903636
C	10.215454	-10.168332	-4.636321
C	10.369257	-14.270088	-1.883363
C	9.814304	-13.554939	-2.947324
C	10.613213	-16.407043	-0.668117
C	10.142457	-15.684306	-1.722686
C	11.039131	-18.532494	0.532236
C	10.395075	-17.839624	-0.504212
C	17.589079	-10.276727	2.418958
O	18.691512	-10.415353	2.941270
O	17.469417	-9.986087	1.106149
C	12.152701	-6.072566	0.029706
O	12.512288	-5.324196	0.937584
O	11.253295	-5.558688	-0.924671
O	15.794096	-7.670465	3.492829
O	18.007951	-12.398474	4.533849
O	17.121841	-13.879745	6.609030
C	16.713118	-18.804487	8.501794
O	16.253356	-19.898889	8.799840
O	17.542381	-18.118942	9.348461
C	14.363525	-20.335208	4.082580
O	13.364952	-21.012326	3.896263
O	15.586789	-20.924149	4.280264
C	9.529757	-18.553254	-1.456300
O	9.183810	-18.215970	-2.575576
O	9.065433	-19.779412	-0.967255
C	8.795897	-14.175651	-3.832682
O	8.780993	-14.137485	-5.052428
O	7.785271	-14.791311	-3.137730
O	11.729220	-8.355040	-4.200218
O	10.885045	-19.841958	0.771882
H	14.683503	-9.378889	-0.255896
H	13.924841	-6.496246	2.041720
H	13.891670	-12.019148	3.598580
H	16.447922	-10.007967	4.171069
H	17.283257	-14.761297	3.698235
H	15.541724	-19.856650	6.397089
H	17.310235	-16.252108	7.865244
H	12.838541	-16.428239	4.107309
H	11.500220	-7.579222	-2.060968
H	12.890102	-10.565654	-3.243219

H	10.421279	-15.450486	1.159282
H	12.622544	-19.760575	2.254058
H	8.997741	-11.914532	-4.974869
H	9.776487	-9.634774	-5.483535
H	9.620403	-16.207267	-2.514932
H	18.383603	-9.967789	0.750704
H	11.167453	-4.623980	-0.646394
H	15.571399	-6.721333	3.467912
H	18.338963	-11.554294	4.118451
H	17.106664	-14.336232	7.473545
H	17.622336	-18.683737	10.143986
H	15.406750	-21.887750	4.253664
H	8.532055	-20.163480	-1.692589
H	7.207061	-15.178221	-3.828637
H	11.221776	-7.964902	-4.936912
H	10.177183	-20.188451	0.171614
O	12.024309	-21.010114	8.887093
H	12.981465	-21.035457	9.071161
O	4.460959	-19.522690	-1.242849
H	4.533477	-19.365757	-2.201264
O	16.120555	-8.669274	7.601103
H	15.769898	-7.779943	7.784761
O	8.907607	-6.825799	-2.175528
H	9.483277	-6.157275	-1.743200

GO TAPP

C	12.861345	-18.519129	1.078631
C	11.959450	-17.513534	0.484087
C	12.720473	-19.824068	0.732736
C	10.883464	-17.982369	-0.500952
C	11.684754	-20.310634	-0.141236
C	10.834770	-19.466812	-0.771252
C	13.968202	-16.602473	2.343050
C	13.797404	-15.800155	1.046053
C	13.949513	-18.125182	2.053801
C	12.444574	-16.117801	0.416924
C	14.892594	-14.506233	3.668453
C	14.729257	-13.714560	2.379749
C	14.625753	-16.003047	3.549909
C	14.070465	-14.301822	1.173326
C	16.173896	-12.609284	4.632959
C	15.339601	-11.692716	3.811821
C	16.103152	-13.974615	4.403420
C	14.645094	-12.226926	2.625501
C	17.101438	-10.696268	5.831457
C	16.466083	-9.800632	4.894614
C	17.006919	-12.055089	5.644899
C	15.685892	-10.263012	3.890105
C	15.201464	-9.355565	2.817895
C	14.638091	-9.910026	1.533020
C	14.538069	-11.421085	1.357475
C	13.355308	-11.917403	0.557800

C	13.288974	-13.389923	0.296238
C	12.351845	-13.847444	-0.596452
C	11.969314	-15.236043	-0.685394
C	11.153598	-15.746003	-1.681619
C	10.777896	-17.134464	-1.751502
C	10.159731	-17.678557	-2.856987
C	9.834971	-19.858096	-1.750418
C	9.557618	-18.972361	-2.758368
C	10.602859	-14.859530	-2.732957
C	10.143216	-15.430756	-4.035790
C	9.974330	-16.939535	-4.162799
C	11.667146	-12.877744	-1.495372
C	10.894037	-13.409593	-2.624451
C	12.689336	-10.972233	-0.351629
C	12.276986	-11.500455	-1.691882
C	13.826222	-9.043941	0.664177
C	12.982796	-9.527950	-0.270975
C	10.392267	-14.663198	-5.272856
C	10.766512	-15.328736	-6.394038
C	10.685891	-17.527297	-5.361625
C	10.959427	-16.758865	-6.442135
C	10.903921	-12.549026	-3.853654
C	10.644441	-13.197051	-5.173261
C	11.416531	-10.561777	-2.530670
C	10.761558	-11.105122	-3.588031
C	12.164107	-8.658568	-1.105723
C	11.403501	-9.138646	-2.178887
C	13.849570	-18.960912	3.340701
O	14.818552	-19.206743	4.054568
O	12.622808	-19.375626	3.641143
C	9.103684	-21.142825	-1.778066
O	8.305926	-21.479823	-2.641936
O	9.365870	-21.952368	-0.705064
O	13.628384	-20.728015	1.231201
O	15.332876	-16.687989	4.489565
O	17.025096	-14.824893	4.866917
O	11.429697	-16.484364	1.382079
O	13.183307	-16.190403	3.477008
O	15.529487	-14.048014	1.236838
C	17.748126	-10.116180	7.012464
O	17.629192	-8.948931	7.372583
O	18.493350	-11.011097	7.731948
C	14.762072	-7.965803	3.241032
O	15.087723	-6.928869	2.706072
O	13.920861	-8.045117	4.302258
C	10.553511	-8.209460	-2.956790
O	9.948208	-8.456514	-3.988378
O	10.425639	-6.945557	-2.394957
C	10.048794	-12.342781	-6.268517
O	8.903946	-12.466394	-6.653219
O	10.914566	-11.417195	-6.748864
O	10.870752	-18.872554	-5.292493
O	9.177807	-14.955943	-3.075775
O	12.005834	-12.910605	-4.758092

O	10.223593	-12.881963	-1.456490
O	12.202449	-7.378497	-0.727122
O	12.010419	-11.424577	0.848596
O	16.007340	-9.405348	1.630547
O	13.845604	-11.874402	3.756521
H	9.933486	-17.771560	0.045822
H	11.603780	-21.387261	-0.301021
H	14.568854	-16.162768	0.341904
H	14.939630	-18.385859	1.638807
H	14.042943	-14.151121	4.279787
H	16.656634	-8.732956	5.012116
H	17.492694	-12.710223	6.372071
H	15.418121	-11.706145	0.755258
H	8.864286	-19.292523	-3.534471
H	8.898377	-17.051266	-4.424454
H	13.221189	-11.649613	-2.252373
H	13.985782	-7.970640	0.759344
H	11.041572	-14.755120	-7.281582
H	11.370208	-17.207185	-7.349569
H	10.192283	-10.481211	-4.267701
H	12.663730	-19.725082	4.565379
H	8.800671	-22.740299	-0.844435
H	13.346819	-21.626297	0.986506
H	15.314706	-17.667568	4.318952
H	17.768584	-14.297998	5.225532
H	18.821608	-10.509472	8.505847
H	13.648722	-7.128288	4.515854
H	9.426200	-6.701690	-2.449777
H	10.401565	-10.881260	-7.390421
H	11.246864	-19.183049	-6.137421
H	11.504435	-6.901837	-1.277045
C	10.463216	-16.733557	5.208987
C	7.775616	-16.163559	1.162079
C	8.580491	-11.301799	1.409984
C	11.311952	-11.880526	5.424847
N	8.333717	-13.776266	1.591715
N	9.108789	-16.043603	3.246139
N	10.679396	-14.256638	5.054617
N	9.839043	-11.991862	3.430250
C	11.192421	-13.229145	5.818578
C	10.802176	-15.462377	5.709784
C	9.755119	-16.980401	4.010377
C	8.553488	-16.731832	2.198897
C	10.746763	-11.352344	4.239312
C	9.523458	-11.092162	2.443519
C	7.967271	-12.537888	1.110900
C	7.603449	-14.774431	0.981995
C	11.619885	-13.816485	7.053603
C	11.387816	-15.172255	6.985369
C	9.639483	-18.302476	3.406546
C	8.895028	-18.149274	2.277586
C	6.671251	-14.122027	0.113074
C	6.887462	-12.766031	0.195318
C	11.067091	-10.031671	3.712897

C	10.306482	-9.867617	2.597319
H	12.026213	-13.258393	7.889223
H	11.576794	-15.911707	7.755589
H	10.105688	-19.212915	3.761517
H	8.638523	-18.919347	1.556657
H	5.916766	-14.633634	-0.473589
H	6.337676	-11.984522	-0.315169
H	11.829389	-9.366003	4.101998
H	10.334051	-9.034924	1.902086
C	10.946967	-17.904451	5.976805
C	12.305711	-18.039646	6.314607
C	10.086226	-18.959432	6.331152
H	13.005755	-17.256031	6.025818
H	9.024451	-18.871534	6.098459
C	12.791030	-19.188321	6.938269
C	10.556060	-20.105820	6.965144
H	13.858179	-19.274389	7.154276
H	9.859096	-20.904256	7.230156
C	11.921841	-20.245034	7.277229
C	7.150748	-17.059513	0.164050
C	6.469357	-18.238811	0.529613
C	7.250809	-16.781769	-1.212725
H	6.331143	-18.462682	1.587566
H	7.782984	-15.892296	-1.544769
C	5.964040	-19.116261	-0.422919
C	6.727561	-17.642010	-2.170246
H	5.440682	-20.020241	-0.104001
H	6.825745	-17.390673	-3.229900
C	6.097018	-18.842850	-1.797780
C	8.250482	-10.168139	0.513710
C	7.963939	-8.879227	1.003014
C	8.245932	-10.346472	-0.881893
H	7.912385	-8.720242	2.080154
H	8.498054	-11.321539	-1.292925
C	7.754720	-7.804755	0.139862
C	7.995984	-9.289986	-1.748664
H	7.565321	-6.809317	0.548257
H	8.033122	-9.455431	-2.825227
C	7.787971	-8.000430	-1.246256
C	12.132701	-10.990118	6.274201
C	13.415671	-11.375407	6.709570
C	11.693566	-9.705455	6.647095
H	13.792936	-12.359915	6.436857
H	10.689188	-9.389425	6.364232
C	14.229736	-10.518287	7.434937
C	12.510426	-8.831395	7.357870
H	15.216938	-10.851561	7.752886
H	12.140444	-7.837545	7.622047
C	13.814016	-9.207529	7.734799
H	9.017107	-13.942669	2.331320
H	10.218396	-14.120147	4.154151
N	12.411449	-21.416622	7.851251
H	11.729825	-21.975735	8.355420
N	14.659949	-8.343565	8.422880

H	15.655733	-8.478486	8.240275
N	7.754170	-6.889376	-2.146670
H	7.340940	-7.129055	-3.049308
N	5.568848	-19.706857	-2.754393
H	5.553348	-20.683386	-2.471005
H	7.277237	-6.076432	-1.755643
H	14.394979	-7.363602	8.386937
H	13.288426	-21.324523	8.355013
H	5.987194	-19.613543	-3.676063

RGO TAPP

C	9.271906	-11.212242	1.185335
C	12.206943	-11.747359	5.086358
C	10.933147	-16.505108	5.408937
C	7.992677	-15.964966	1.526349
N	11.385173	-14.096028	4.975860
N	10.628078	-11.906491	3.163976
N	8.862010	-13.629201	1.585043
N	9.572718	-15.814203	3.443723
C	7.994943	-14.610884	1.140836
C	8.580888	-12.421210	0.975780
C	10.291659	-11.034145	2.155125
C	11.589462	-11.259692	3.908200
C	8.812240	-16.518979	2.539945
C	10.144782	-16.762939	4.260266
C	11.430970	-15.231325	5.758985
C	12.022459	-13.047070	5.603370
C	7.113049	-13.979551	0.206078
C	7.463698	-12.653745	0.109823
C	11.108261	-9.832471	2.224272
C	11.917360	-9.973350	3.311507
C	12.474451	-13.542140	6.868273
C	12.110793	-14.866715	6.965121
C	8.968407	-17.953381	2.743855
C	9.805598	-18.104671	3.808580
H	6.303410	-14.483842	-0.307082
H	6.976379	-11.890357	-0.484168
H	11.092775	-9.006020	1.524504
H	12.680008	-9.284230	3.648621
H	12.977423	-12.942737	7.617923
H	12.265943	-15.531529	7.806576
H	8.537150	-18.733203	2.125643
H	10.201180	-19.029655	4.214120
C	8.907991	-10.040731	0.358819
C	8.743843	-10.130081	-1.034364
C	8.744745	-8.766072	0.940227
H	8.867714	-11.093407	-1.523315
H	8.804279	-8.668259	2.024171
C	8.484616	-9.006920	-1.810873
C	8.514919	-7.634331	0.171080
H	8.385092	-9.113162	-2.891593
H	8.412895	-6.661073	0.654628

C	8.404874	-7.731450	-1.227130
C	13.175970	-10.877964	5.796488
C	12.901275	-9.532576	6.105409
C	14.453591	-11.364407	6.136134
H	11.908483	-9.133532	5.896770
H	14.699285	-12.403402	5.922500
C	13.864356	-8.705189	6.671925
C	15.426346	-10.547898	6.698320
H	13.616134	-7.667810	6.905536
H	16.413347	-10.961727	6.915477
C	15.154294	-9.192044	6.960211
C	11.232240	-17.625880	6.326808
C	10.215352	-18.492404	6.775558
C	12.535893	-17.869805	6.795536
H	9.189339	-18.314490	6.452446
H	13.349606	-17.229905	6.457197
C	10.485611	-19.551802	7.633143
C	12.818485	-18.922609	7.653049
H	9.671897	-20.196983	7.971182
H	13.842249	-19.105463	7.979660
C	11.798708	-19.791276	8.083988
C	7.072401	-16.877349	0.808944
C	7.056629	-16.958779	-0.593586
C	6.186524	-17.711568	1.517478
H	7.742029	-16.344662	-1.175794
H	6.168803	-17.658198	2.606932
C	6.206253	-17.835457	-1.259474
C	5.326100	-18.584303	0.861754
H	6.244790	-17.895505	-2.349992
H	4.636464	-19.204584	1.438608
C	5.323015	-18.665944	-0.544713
H	10.861056	-14.016968	4.102070
H	9.520381	-13.730086	2.361262
N	16.113510	-8.373604	7.548557
H	15.998772	-7.376825	7.392344
H	17.076081	-8.677925	7.436175
N	8.167866	-6.597874	-2.003674
H	8.398761	-6.707176	-2.987304
H	8.567006	-5.744413	-1.622087
N	4.427894	-19.500866	-1.208611
H	4.079086	-20.293257	-0.678560
H	4.669485	-19.739348	-2.165180
N	12.083024	-20.817195	8.975219
H	13.058593	-21.095636	9.030355
H	11.438776	-21.601182	8.981501
C	15.171282	-9.792714	2.551261
C	14.345691	-10.456163	1.593836
C	15.054084	-8.423131	2.663492
C	13.788786	-9.631901	0.488640
C	14.123504	-7.641868	1.900295
C	13.455735	-8.189427	0.833670
C	15.943807	-12.053943	3.399810
C	14.595033	-12.526175	2.895902
C	16.249328	-10.563569	3.259864

C	14.120535	-11.816624	1.653117
C	16.475312	-14.326663	4.314737
C	15.185665	-14.826485	3.686699
C	16.765614	-12.869403	4.098216
C	14.419516	-14.015369	2.856655
C	16.327307	-16.219018	5.908582
C	15.551360	-16.917972	4.883366
C	16.605160	-14.867885	5.714709
C	14.890285	-16.181899	3.902230
C	16.394357	-18.262177	7.226689
C	15.725335	-18.970339	6.183178
C	16.689135	-16.920289	7.090285
C	15.289174	-18.332547	5.036910
C	14.476666	-19.001869	4.024240
C	13.683546	-18.260059	3.149379
C	13.656615	-16.755995	3.288159
C	13.129783	-15.983368	2.108731
C	13.433540	-14.583029	2.000003
C	12.840590	-13.822462	0.940917
C	13.245430	-12.457669	0.727415
C	12.697665	-11.733355	-0.368364
C	12.879394	-10.331831	-0.457122
C	12.249657	-9.560035	-1.443828
C	12.552605	-7.513906	-0.076113
C	12.054454	-8.197183	-1.198854
C	11.767539	-12.362134	-1.275119
C	11.272744	-11.677163	-2.381631
C	11.873755	-10.317642	-2.706515
C	11.879228	-14.412725	0.073896
C	11.356484	-13.711938	-1.016501
C	12.243077	-16.599428	1.264077
C	11.329844	-15.776741	0.408541
C	12.796535	-18.816561	2.196058
C	12.051355	-18.017956	1.335769
C	10.286485	-12.261942	-3.237773
C	9.757673	-11.494948	-4.281586
C	11.164269	-9.578930	-3.795567
C	10.191928	-10.172494	-4.544426
C	10.425051	-14.348727	-1.915288
C	9.859016	-13.615679	-2.959953
C	10.682221	-16.506021	-0.741710
C	10.217213	-15.768880	-1.788874
C	11.083287	-18.640617	0.451022
C	10.455142	-17.938765	-0.590181
C	17.578516	-10.409050	2.518161
O	18.677261	-10.531449	3.054973
O	17.472108	-10.147585	1.198658
C	12.164751	-6.133053	0.163048
O	12.525362	-5.413900	1.095131
O	11.297113	-5.613093	-0.789379
O	15.868722	-7.779922	3.551084
O	17.963621	-12.519250	4.639045
O	17.009621	-14.029209	6.687079
C	16.699216	-19.002443	8.464746

O	16.277205	-20.118557	8.733059
O	17.497370	-18.308254	9.336398
C	14.443076	-20.483645	3.967082
O	13.474797	-21.186586	3.719163
O	15.674792	-21.045829	4.195680
C	9.593804	-18.638558	-1.553766
O	9.246553	-18.284065	-2.667600
O	9.134798	-19.878805	-1.091534
C	8.855473	-14.229799	-3.867052
O	8.845584	-14.156722	-5.085608
O	7.856884	-14.884227	-3.193879
O	11.689367	-8.350348	-4.076788
O	10.904039	-19.946381	0.694607
H	14.693463	-9.480780	-0.162534
H	13.976030	-6.587285	2.135875
H	13.865325	-12.203644	3.673870
H	16.425784	-10.145109	4.265848
H	17.280254	-14.874928	3.767745
H	15.535206	-20.027673	6.343596
H	17.246730	-16.427921	7.890455
H	12.841340	-16.598668	4.047585
H	11.447638	-7.624546	-1.894849
H	12.864378	-10.559714	-3.175479
H	10.473296	-15.579978	1.097835
H	12.676034	-19.894389	2.154671
H	9.006790	-11.930875	-4.935265
H	9.752533	-9.630071	-5.386769
H	9.701166	-16.279810	-2.592537
H	18.389812	-10.135329	0.852456
H	11.184733	-4.686540	-0.495560
H	15.647230	-6.831018	3.523352
H	18.302816	-11.679313	4.222746
H	16.962982	-14.500071	7.542753
H	17.588132	-18.889894	10.118354
H	15.521407	-22.011889	4.129893
H	8.606316	-20.245469	-1.828933
H	7.291127	-15.273749	-3.893596
H	11.253167	-8.005005	-4.877978
H	10.205672	-20.285588	0.079159
